# A multi-modal machine learning approach to detect extreme rainfall events in Sicily

**DOI:** 10.1038/s41598-023-33160-9

**Published:** 2023-04-16

**Authors:** Eleonora Vitanza, Giovanna Maria Dimitri, Chiara Mocenni

**Affiliations:** Department of Information Engineering and Mathematics, University of Siena, Via Roma, 56, 53100 Siena, Italy

**Keywords:** Climate sciences, Environmental sciences, Environmental social sciences

## Abstract

In 2021 almost 300 mm of rain, nearly half of the average annual rainfall, fell near Catania (Sicily Island, Italy). Such events took place in just a few hours, with dramatic consequences on the environmental, social, economic, and health systems of the region. These phenomena are now very common in various countries all around the world: this is the reason why, detecting local extreme rainfall events is a crucial prerequisite for planning actions, able to reverse possibly intensified dramatic future scenarios. In this paper, the Affinity Propagation algorithm, a clustering algorithm grounded on machine learning, was applied, to the best of our knowledge, for the first time, to detect extreme rainfall areas in Sicily. This was possible by using a high-frequency, large dataset we collected, ranging from 2009 to 2021 which we named RSE (the Rainfall Sicily Extreme dataset). Weather indicators were then been employed to validate the results, thus confirming the presence of recent anomalous rainfall events in eastern Sicily. We believe that easy-to-use and multi-modal data science techniques, such as the one proposed in this study, could give rise to significant improvements in policy-making for successfully contrasting climate change.

## Introduction

Is it possible to detect extreme rainfall events areas by clustering spatio-temporal data? The intensification of weather extremes, which is dramatically changing the climate scenario worldwide, is currently thought to be as one of the most important factors related to green-house effect and climate change^1–8^. The increase in the frequency and intensity of daily temperatures has contributed to a widespread escalation of daily precipitation^9,10^. Moreover, severe weather and climate events, interacting with exposed and vulnerable human and natural systems, can lead to disasters which require an extraordinary adaptation ability^2^. It is therefore mainly for this reason that, nowadays, the study of climate change is not only about temperature increase, but it also focuses on catastrophic rainfall extreme events and drought^7,11^. The concept of *extreme* precipitation and its changes in response to warming are well described in^12^. For this reason, the scientific community faces an increasing demand for regularly updated estimations of evolving climate conditions and extreme weather events^1,11,13^. Moreover, a correlation between changes in heavy precipitation and landslides in several regions has been found in^2^. More specifically, it is possible to identify 3 examples of extreme weather events, that have raised the question of a potential link to climate change: more intense precipitation events, increased summer drying over most mid-latitude areas and increase in tropical cyclone peak winds intensities^14^. These results show that rainfall extreme events are related to climate change^15^ and represent the triggers of a chain of reactions involving several human activities. The change in temperatures will, in fact, have serious long-term effects^16–18^, although extreme rainfall events will also cause a short-term danger to the environment and the population^19^. In more recent years several extreme events all over the world caused large losses of lives, as well as a tremendous increase in economic losses from weather hazards^20^. Such disasters have forced public opinion to consider climate change as the main cause of these events^21^ and to deeply analyse the economic consequences of climate change in terms of investments and productivity^22,23^. A relevant example of this regards wine industry. For instance, in the past two decades, Sicilian winemakers have enhanced the biological production of wine all around the island, especially on the slopes of Mount Etna. Although wine is not essential to human survival, it is an important product of human ingenuity and its economy is rapidly growing^24^. Agricultural activities depend on climate and are interconnected to weather changes. Any shift in climate and weather patterns may potentially affect the entire local wine industry^25^ and the stability of many crops, thus undermining the related economies^23^. Any shift in climate and weather patterns may potentially affect the entire local wine industry^25^. Abnormal climate changes might also undermine the stability of crops and might be critical for the related economy^23^. Considering all of these aspects, in Mediterranean areas, rainfall is probably the most important climatic variable due to its manifestation as a deficient resource (dryness) or a catastrophic agent, such as water bombs^26^. Therefore, many challenges arise during the measurement of the precipitation. For instance, in situ measurements are especially affected by wind effects on the gauge catch, particularly for snow but also for light rain^16^. Moreover, to reduce this uncertainty, it is crucial to analyze spatio-temporal data in the most efficient way^27,28^.

In this regard, over the last decades scientists conducted several studies on rainfall time series. These studies investigated potential trends in different rainfall indicators, such as total and maximum annual precipitation and mean daily intensity^29–31^. A tendency toward higher frequencies of heavy and extreme rainfalls emerged for some areas^32^. In most of these areas, an increase in total precipitation has also been observed, for instance in^26^, thanks to the analysis of 247 stations over the 1921–2000 period. However, the correlation between the increase of total precipitation and extreme events is not always clear, as in other areas (i.e. Italy) several authors have observed an increase in heavy precipitation, together with a tendency towards a decrease in the total amount of precipitations^33^. Among the studies mentioned, a few of them were specifically focused on the Mediterranean areas, given their peculiar climate, which is affected by interactions between mid-latitude and tropical processes, lying between the arid climate of North Africa and the temperate and rainy climate of central Europe. For these reasons, even relatively minor modifications of the general circulation can lead to substantial changes in the Mediterranean climate^29,30–35^, including rainfall frequency^36^, thus making these areas vulnerable to climatic changes and in particular to catastrophic precipitations.

In this setting, scientists analysed the region of Sicily to identify climate change signals, as for instance in^37^. In most of those studies, the authors analysed annual, seasonal and monthly rainfall data in the entire Sicilian region, showing a global reduction of total amount of annual rainfall^37^. For example, in^29^ the annual maximum rainfall for fixed time duration of 1, 3, 6, 12 and 24 h, and the daily rainfall series recorded from 1956 to 2005 in approximately 60 stations were analyzed using the non-parametric Mann–Kendall test^38,39^.

Results of this study, confirmed an increasing trend for rainfall of short duration, in particular for the 1 hour rainfall length. On the other hand, time-persistent rainfalls exhibited a decreasing course^38,39^. In particular, heavy-torrential precipitation have been reported to be more frequent at a regional scale, while light rainfall have shown negative trends at some sites. In^40^ the presence of linear and non-linear trends in 16 series from rain gauge stations, mostly placed in the eastern Sicily, was studied. The results indicated a different behaviour according to the time scale: for short duration, historical series generally presented increasing trends, that switched to decreasing for longer time courses.

A total of 67 sites of daily precipitation records over the 1951–1996 period in Italy were also analyzed in^33^ considering seasonal and yearly total precipitation, number of wet days and precipitation intensity with the aim of evaluating the trends both from the single-station records, and for larger areas by using averaged series. Results showed that the trend for the number of wet days in the year was significantly negative throughout Italy, particularly stronger in the north than in the south, especially in winter. A tendency towards an increase in precipitation intensity, which was globally less strong and significant than the decrease in the number of wet days was also found.

In^41^ the authors identified the presence of homogeneous areas over Sicily using the Regional Frequency Analysis (RFA), which is a procedure estimating the frequency of rare events at one site by using data from several sites^42^, used frequently in the analysis of environmental data^43^. They also developed Principal Component Analysis (PCA) followed by a clustering analysis, performed by applying the K-Means method, to identify regional groups, starting from annual maximum series for rainfall duration of 1, 3, 6, 12 and 24 h over about 130 rain gauges.

One of the most interesting papers studying different rainfall time series in Sicily is^32^, where the authors investigated temporal changes in extreme rainfall by performing a regional study. In particular, a regional frequency analysis based on L-moments approach^44^ was applied to 1, 3, 6, 12 and 24 h annual maxima rainfall (AMR) series grouped per homogeneous regions, identified through a hierarchical cluster analysis^45^. Changes were investigated in a long-term dynamic (from 1928 to 2009) with special reference to the last forty years. The study^32^ detected an increasing trend on rainfall extreme events between 2003 and 2009 with several heavy localized storms all over Sicily and a remarkable tendency towards more intense storm events during the 2000’s affecting mainly the outer western part of the region. On the contrary, the increasing trend in extreme rainfall detected in eastern Sicily, has been considered only apparent, as related to a few severe local storms.

In our work we present for the first time a multi-modal spacial and temporal clustering analysis on rainfall data over Sicily, performed using the Affinity Propagation clustering algorithm^46^. The novelties are manifold. First of all, we collected a new dataset, which we named RSE (Rainfall Sicily Extreme), offering an original perspective on extreme events happening from 2009 up to 2021, witnessed by the alarming violent rainfall events that occurred in East Sicily at the end of 2021^47,48^. Moreover, the analysis was performed directly on the whole time series, without defining any specific statistic indicator or feature extracted from the data. In this way we avoid the risk of introducing any bias or a priori assumptions, such as homogeneity of the whole Sicily or its sub-regions, and the need of performing data dimensionality reduction. Additionally, the data preprocessing phase allowed us to remove data inconsistencies. Finally, the Affinity Propagation algorithm, successfully used in other contexts^49–52^, is here applied to climate data for the first time.

Differently from^32^ or^35^, in our study clustering is not only used for identifying homogeneous sub-regions, but also to detect critical rainfall sites. Moreover, while in^32^ the authors focused on finding long-term trends, we concentrated our attention on short-term changes between 2009 and 2021, analyzing high-frequency data, so as to obtain clusters specifically related to extreme events.

Based on the RSE dataset, we faced several steps:

- We clustered regions and detect *extreme* sites according to rainfall data observations.
- We used a multi-modal approach to merge both geographical and temporal information.
- We defined rainfall indicators to further validate the clusters and their meaning.
- We detected an increasing trend on extreme events in East Sicily, in agreement with the results of the state of the art in^32^.

**Figure 1:**
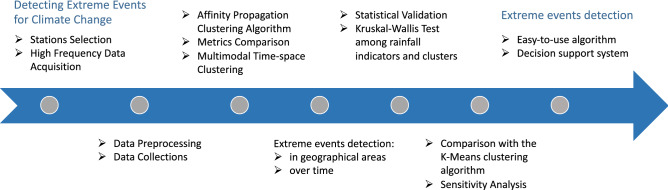
Flowchart of the methodology and the timeline used in this study: data collection, clustering and statistical validation, comparison with other algorithms, conclusions and policy implications.

Figure 1 shows the corresponding methodology flowchart.

The paper is structured as follows: in “RSE: the rainfall Sicily extreme dataset” section the regional dataset used in the analysis, including the data pre-processing, is presented. In “Methods” section the methods applied in the study, in particular the adopted clustering algorithm, and the statistical validation methods, are introduced. In “Results and discussion” section we report the discussion of the results, concerning each analyzed variable, and the most relevant conclusions drawn. Furthermore, we report in the supplementary material the analysis concerning the annual histograms of specific rain gauges and local data plots at different levels, as well as the complete annual clustering results.

### RSE: the rainfall Sicily extreme dataset

**Table 1:** Dataset collections.

Name	Description	# Datasets per station	# Total datasets	# Records per dataset
C.*A*	Annual collection	13	442	52,560*
C.*B*	Annual collection Weekly mean	13	442	53
C.*C*	Full collection	1	34	683,713
C.*D*	Full collection weekly mean	1	34	679
C.$A_{s}$	Single stations collection	13	13	52,560*
C.$B_{s}$	Single stations collection weekly mean	13	13	53

The number of considered stations is 34, except for the Single stations Collections. * 52704 for leap years.

The dataset used in this analysis consists in geographical rainfall records with a 10 minutes periodicity from 2009 to 2021, provided by SIAS, the Servizio Informativo Agrometeorologico Siciliano^53^. The dataset together with the code is available at the following GitHub Repository^54^.

The most common rainfall measurement gathered from the database is the number of millimeters (mm) of rain in a given period. Accordingly, six collections were considered, as described in Table 1. C.*A* and C.*B* contain 13 datasets per station—one per year—with the original data and the weekly mean data, respectively. C.*C* and C.*D* include one full dataset per station - involving all the records from 2009 to 2021—with the original data and the weekly mean data, respectively. C.$A_{s}$ and C.$B_{s}$ are subsets of C.*A* and C.*B*, respectively, since one station per time is considered, so that each of them includes 13 datasets.

### Data preprocessing

We will now describe the initial data selection process, obtained through the analysis of annual data. On the basis of an initial graphical analysis reported in the SI document, we decided to select the most extreme stations. A station is considered *extreme* if it is possible to observe a high amount of rain in a relatively short time interval. We implemented this concept of “extremeness” using the following strategy.

First, we considered the following data for all the 96 available stations in Sicily and for all the years:

- The total annual precipitation in mm (*tot*).
- The percentage of rainy days over the year (*rd*), measured as number of days with more than 1 mm of rain.
- The mm of rain during the rainiest day in the year (*dmax*).

Afterwards, a selection strategy has been applied. Extreme rainfall events are generally characterized by the increasing of either drought and/or excessive wetness^26^. The logical rule below highlights precisely such characteristics:

1. Fix a station.
2. Compute $\mu _{1}$: the mean over years of the *rd* annual indicator.
3. Compute $\mu _{2}$: the mean over years of the *dmax* annual indicator.
4. Fix a year *y*.
5. If the *rd* value in the year *y* is less than $\mu _{1}$ and the *dmax* value in the year *y* is grater than $\mu _{2}$, then the year *y* is considered as *extreme*. Otherwise no.

**Figure 2:**
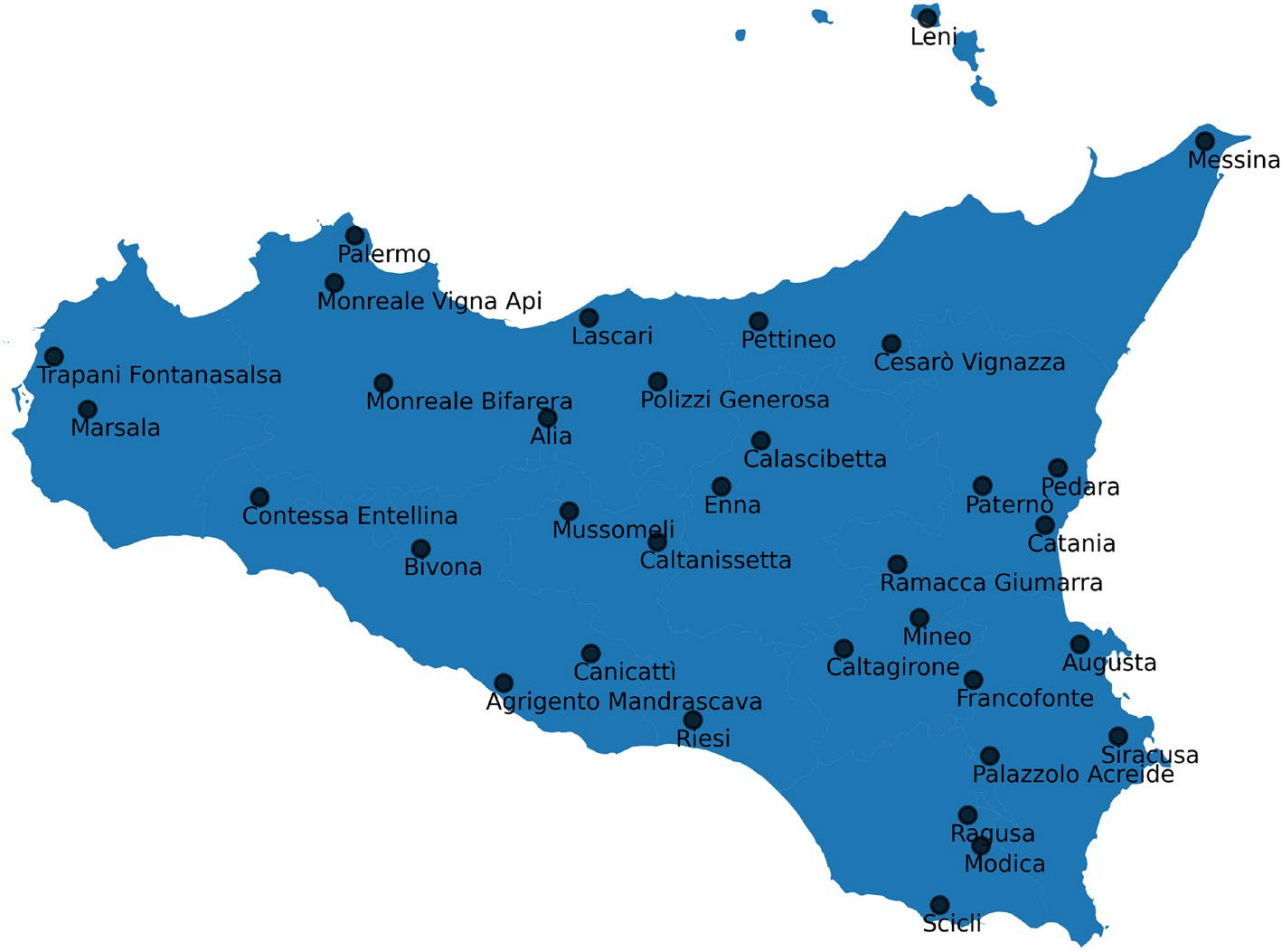
Location of rainfall gauging stations in Sicily.

Since the procedure works year by year, we selected the stations satisfying the *extreme events* detection rule for at least 3 years (the stations respecting this condition for at least one year were 85 out of 96, almost all). In this way, we obtained 32 stations out of the 96 rain gauges. Furthermore, we decided to include all of the provincial capitals in the region, thus obtaining the 34 stations shown in Fig. 2.

After the selection, we observed rainfall data time series, by fixing a station and using full, annual, and monthly data plots, as well as mean data graphics (all details regarding these initial observations are reported in the SI document). This preliminary analysis lead to different reasoning. The full plots proved the necessity of quantifying and understanding variation in the stations time series behavior. In contrast, the annual plots showed a typical seasonality pattern. Moreover, the graphics observation led to the idea of comparing annual time series. Finally, a similar reasoning has been done with regard to the monthly view.

All of the above considerations suggested us to highlight the differences and the similarities both among stations and years, in order to identify multi-modal (geographical and historical) rainfall changes. Instead of performing classical time series analysis, we proceeded by applying the suitable clustering algorithms described in the following sections.

## Methods

Clustering is an unsupervised machine learning methodology^55,56^. Its goal is to detect groups of observations sharing similar characteristics. More precisely, it consists in the partitioning of a dataset into subsets, so that the data in each subset are characterized by a higher similarity than elements in different sets, according to some defined distance measure.

Two main types of clustering techniques can be defined: methods in which the number of clusters needs to be established a priori, as, for instance, the K-Means algorithm^57^, and algorithms in which, instead, the number of clusters is inherently estimated during the optimization phase, such as the Affinity Propagation^46^. The last one has been used in this work, for several reasons. Firstly, no prior knowledge on the number of expected clusters was available. Secondly, there were no clues about the possible initial centroids. In any case, a detailed comparison of the two algorithms is given in the “Comparison between affinity propagation and K-means” section below.

### Affinity propagation algorithm

Affinity Propagation (AP), introduced by Frey and Dueck in 2007^46^, and its extension to Hierarchical Affinity Propagation^58^, are nowadays becoming extremely popular due to their simplicity, general applicability, and performance and have been succesfully applied to several contexts in research^49–52^.

AP takes as input the measures of similarity between pairs of data points, and simultaneously considers all of them as potential exemplars. The number of clusters does not need to be defined in advance, indeed the algorithm is based on the hypothesis that the so called “real-valued messages” are exchanged between data points until a high-quality set of exemplars, together with the corresponding clusters, gradually emerges. Given that no assumption on the number of clusters was requested in our case, AP has been a natural choice.

The algorithm requires two inputs parameters^46^:

- Similarities *s*(*i*, *k*) between data points, representing how similar a point is to be another one’s exemplar. If there is no similarity between two points, as in this case they cannot belong to the same cluster, this similarity can be omitted or set to $-\infty$ depending on the implementation.
- Preferences *s*(*k*, *k*), indicating each data point’s suitability to be an exemplar. Since some prior information which points could be favored for being an exemplar can be available, it can be represented through preferences.

Similarity is usually defined starting from the negative Euclidean distance or the Pearson correlation coefficient, depending on the considered situation.

If all data points are supposed to be equally suitable as exemplars, the preferences should be set to a common value, such as for example the median of the input similarities, thus resulting in a moderate number of clusters, or their minimum, thus resulting in a small number of clusters^46^. In this work we initialized the preferences to the median and the availabilities to zero, $a(i,k) = 0$. Each iteration step of the optimization performance is composed by 2 main message-passing steps:

1. Computing responsibilities:
  1 $$\begin{aligned} r(i, k) \leftarrow s(i, k)-\max _{k^{\prime } \text{ s.t. } k^{\prime } \ne k}\left\{ a\left( i, k^{\prime }\right) +s\left( i, k^{\prime }\right) \right\} , \end{aligned}$$
  where *s*(*i*, *k*) and $s(i,k')$ are similarities, while $a(i,k')$ are availabilities.
2. Computing availabilities
  2 $$\begin{aligned} a(i, k) \leftarrow \min \left\{ 0, r(k, k)+\sum _{i' \text{ s.t. } i' \notin \{i, k\}} \max \{0, r(i', k)\}\right\} , \end{aligned}$$
  where *r*(*k*, *k*) are the self-responsibilities, while $r(i',k)$ are general responsibilities. To limit the influence of strong incoming positive responsibilities, the total sum is lower bounded, so that it cannot be negative.

The“self-availability”, *a*(*k*, *k*) is updated differently, as follows:

3 $$\begin{aligned} a(k, k) \leftarrow \sum _{i' \text{ s.t } i' \ne k} \max \{0, r(i', k)\}. \end{aligned}$$

The way for calculating how suitable a point is for being an exemplar is that it is favored more if the initial preference was higher, but the responsibility gets lower when there is a similar point that considers it as a good candidate, so there is a “competition”between the two, until one of the two options is chosen in some iteration. The above procedure may be terminated after a fixed number of iterations, after changes in the messages fall below a threshold, or after the local decisions stay constant for a given number of iterations^46^.

### Statistical validation

To assess the presence of statistical differences between 2 communities we made use of the well known Kruskal–Wallis test^59–61^.

#### Kruskal–Wallis test

It is a non parametric statistical test that assesses the differences among three or more independently sampled groups^62^. Kruskal–Wallis test is used to determine whether or not there is a statistically significant difference between the medians of three or more independent groups. It does not assume normality in the data and is much less sensitive to outliers than the standard analysis of variance (ANOVA)^63^. The test is based on the null hypothesis $H_{0}$^64^, which allows one to state whether the considered samples are realizations of identical populations. The application of the test returns a p-value which confirms or rejects the null hypothesis. If $p < 0.05$, then the null hypothesis is rejected, on the contrary, if $p \ge 0.05$, then the null hypothesis is confirmed^63^. The related p-value for the test is computed using the assumption that *H* has a $\chi ^{2}$ distribution.

### Experiments

This section explains the experimental procedure followed to design and apply the clustering algorithm. Global and local clustering analysis have been preformed by means of high frequency (measurements collected every 10 minutes) and weekly averaged data. The reason of this choice lied in the need to reduce the dataset dimension for minimizing sensitivity to outliers and oscillations in the original time series of observations.

Two main streams of experiments were performed:

1. *Geographical (or spacial) clustering.* It consists of grouping similar geographical stations together along different time horizons.
2. *Local (or temporal) clustering.* It consists of grouping similar years together on each single location.

For the first category, we ran the algorithm four times, according to the four Collections of datasets C.*A*, C.*B*, C.*C* and C.*D* described in Table 1. In contrast, the second category involves C.$A_{s}$ and C.$B_{s}$ of Table 1. The Affinity Propagation algorithm has been implemented in Python programming language (version 3), making use of with the Scikit-learn library (V. 1.0.2), which is a free software machine learning library for Python^65^, designed to inter-operate with the Python numerical and scientific libraries NumPy (V. 1.21.4)^66^, SciPy (V. 1.8.0)^67^ and Pandas (V. 1.3.5)^68^. The algorithm was implemented using default hyper-parameters. For instance, *convergence_iter* was set to 15, that is the number of iterations with no change in the number of estimated clusters, that stops the convergence. Moreover, the *preference* value was set to the median of the input similarities.

#### Metrics for affinity propagation

Two different metrics of similarities were used in the Affinity Propagation algorithm: the Euclidean Metrics and the Correlation Metrics. We present in the following subsections the results obtained for both.

##### Euclidean Metrics

 We first conducted clustering using the Euclidean *affinity* metric, which resulted in a principal large cluster and few smaller communities consisting in one element each. For this reason, we decided to apply an iterated version of the AP algorithm in order to detect new geographical clusters, at first glance hidden by the anomalies. To this aim, the AP algorithm based on a particular multi-step structure was implemented as follows:

1. AP is applied to the whole considered collection of datasets.
2. The exceptions found at level one from the data are removed, and the AP algorithm reiterated over the remaining datasets.
3. The process is repeated from Step 1.

##### Correlation Metrics

 On the basis of the theoretical arguments reported in the *Affinity Propagation algorithm* subsection, the Correlation distance was also chosen as a *affinity* metric for a second exploration analysis. In this case no multi-step procedure was needed.

#### Clusters validation procedure

In order to understand the rainfall phenomena that mostly characterize the clusters, several rainfall indicators over the time series were introduced according to^69^, as reported in Table 2.

We assembled the original 10 minutes records according to specific needs: naturally an *hour* data includes six consecutive records summed up together, whereas a *day* consists of the sum of 144 consecutive data. We also computed the total number of rainy hours, where one hour is considered“rainy”if its amount of rain is higher than zero. Hence, some of the introduced indicators are the percentages of light (*l*), moderate (*m*), and heavy (*h*) rainy hours over the total. Moreover, we considered the absolute number of violent rainy hours *v*, which is not expressed in percentage since it represents very rare events.

The Kruskal–Wallis test was then applied to the indicators, in order to understand which of them better characterize clusters. To this aim, the SciPy scientific library has been used. Indeed, it provides algorithms for many classes of problems, extends standard tools of array computing, wraps up highly-optimized implementations, is easy to use, and enlarges NumPy^67^.

The Kruskal Wallis test was applied to each indicator and to all the experiments described above, according to the following logical evaluation steps:

1. Fix an indicator *i*.
2. Run the clustering algorithm.
3. Create an array *k* with one element for cluster. Every element of *k* is in turn an array $a_{c}$, containing the indicator values of the stations belonging to that cluster.
4. Run the Kruskal–Wallis test on *k*.
5. If the *p* value is less than 0.05: *i* is considered as characterizing for the clusters. Otherwise not.

**Table 2:** Description of the indicators.

Variable	Indicator	Description
*wh*	Wet hours (%)	Percentage of rainy hours over the total number of hours
*mh*	Maximum per hour	Maximum amount of rain of the data series grouped by hours
*i*	Intensity (mm/h)	Quotient between the total amount of rain and the number of wet hours
*t*	Total rain	Total amount of rain in the time series
*mv*	Maximum daily variation	Maximum rainfall variation between two consecutive days over the total time series
*wd*	Wet days (%)	Percentage of rainy days over the total number of days
*md*	Maximum per day	Maximum amount of rain of the data series grouped by days
*l*	Light rain (%)	Percentage of light (0–2.5 mm) rainy hours over the total number of rainy hours
*m*	Moderate rain (%)	Percentage of moderate (2.6–7.5 mm) rainy hours over the total number of rainy hours
*h*	Heavy rain (%)	Percentage of heavy (7.6–50 mm) rainy hours over the total number of rainy hours
*v*	Violent rain	Number of violent ($>50$ mm) rainy hours in the time series. It was not reported in percentage since it represented very rare events

### Comparison between affinity propagation and K-means

To further validate our methodology, it was decided to carry out a detailed report comparing the AP and the K-Means algorithms. The comparison was conducted on the collections C.*C* and C.*D* of Table 1. Initial experiments were made by fixing both the number of clusters and the initial centroids in the K-Means algorithm, basing our choice on the AP results. A sensitivity analysis was then performed by varying the initial centroids, based again on the results achieved with the AP algorithm. The Jaccard score^70^ between each new experiment and the reference AP results was computed. It is a statistical index used to compare the similarity and diversity of sample sets^70^. We used it to quantify the differences between two experiments A and B, by analyzing the composition of the corresponding clusters in the two cases. The Jaccard score is defined as the size of the intersection divided by the size of the union of the sample sets and it ranges in the interval [0, 1]:

4 $$\begin{aligned} J(A, B)=\frac{|A \cap B|}{|A \cup B|} \end{aligned}$$

Furthermore, for each of those experiments, the cluster validation procedure was carried out, computing the p-values and finding the characterizing indicators for the clusters, among those in Table 2.

A second set of 200 K-Means experiments per collection was conducted fixing the number of clusters and randomly varying the initial centroids. The results of all the experiments are reported in “K-means results” section.

## Results and discussion

In this section we report the main results obtained from the study for both geographical and single station investigations. Additional details on the whole dataset results are reported in the supplementary information document.

### Geographical investigation

**Figure 3:**
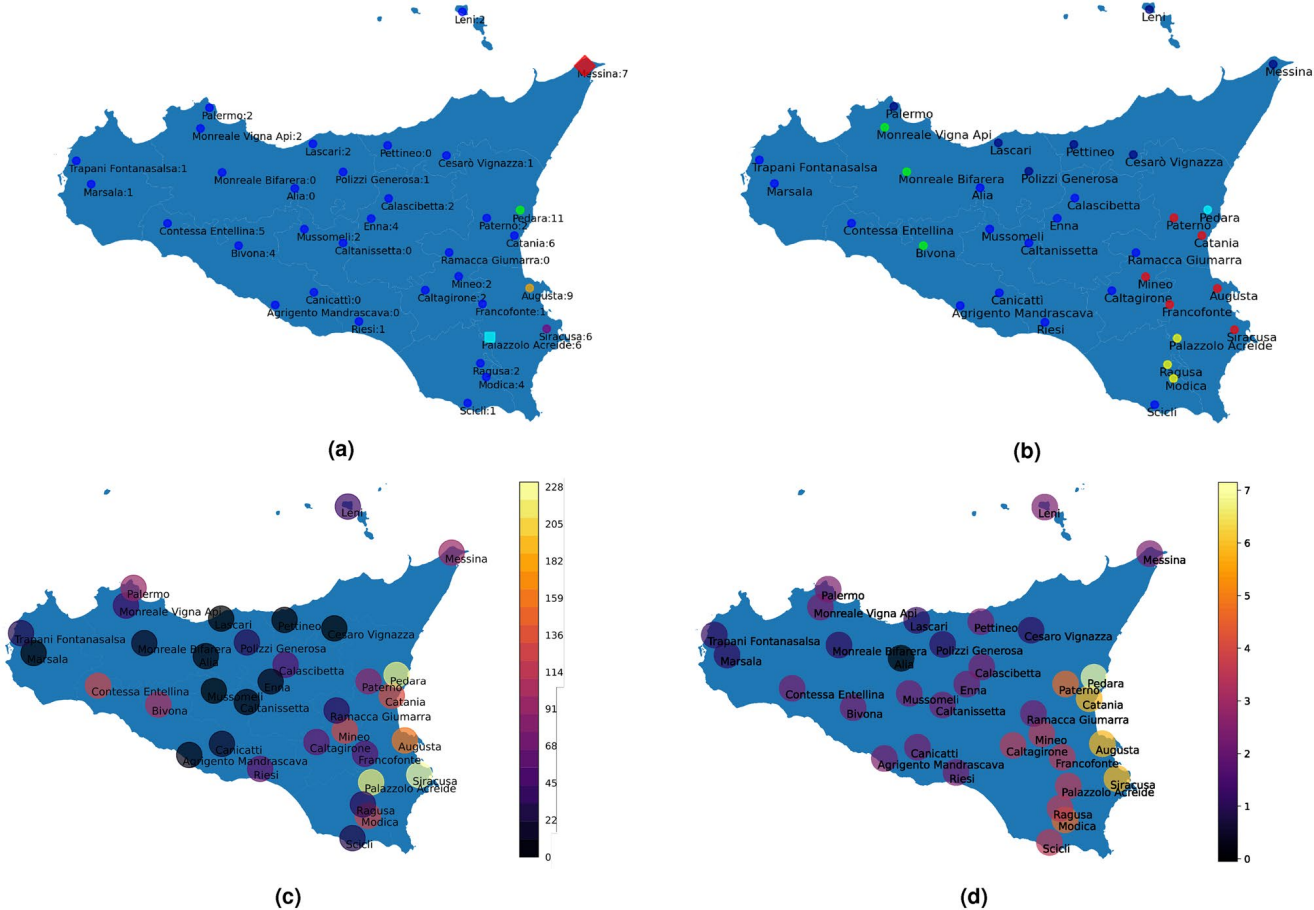
Full case—Euclidean metrics. In (**a**, **b**) different colors represent different clusters, both in the maps and in the histograms. Square and diamond points represent results from, respectively, the second and the third iteration of the algorithm. (**a**) C.*C*. The principal cluster is reported in blue. The numbers indicate how many times the stations has been clustered as anomalous in the annual case. (**b**) C.*D*. The five main clusters are reported in green, blue, dark blue, red and yellow. (**c**) C.*C*. Maximum per day (*md*) heatmap. (**d**) C.*D*. Heavy rain (%) (*h*) heatmap.

Results of this set of experiments are visualized in the Sicily map of Fig. 3, where the 34 stations with names or symbols coloured according to their relative clusters are drawn. When the multi-step version of the algorithm is applied, different shapes for the points are used. Specifically, circle, squares and diamond markers represent clusters resulting from the first, second, and third iterations, respectively.

#### Annual clustering

The results of the geographical clustering year by year for C.*A* and C.*B* (Table 1), both with the Euclidean and Correlation similarities are included in the SI document. Additionally, a video showing the clustering results proceeding in years is available for each collection. We hereby report the main results drawn from the several performed experiments:

- Euclidean metrics—C.*A* (Video_1): in this case the annual results consist mostly of a principal cluster (at most two) and some anomalous stations. Proceeding in the years, a flow in anomalies that goes from western to eastern Sicily is detectable. We claim that anomalous clusters are more susceptible to extreme events. In fact, since them change drastically in intensity according to the specific location, this legitimizes the algorithm in finding exceptions, namely only one station in one cluster. In order to validate this observation, a case by case analysis has been carried out and reported in the SI document for the year 2021.
- Euclidean metrics—C.*B* (Video_2): the reduction in the dataset size led the clusters to be more uniform and referred to geographical divisions. However, there are some exceptions, mainly in the South-East Sicily, and in the neighbourhood of *Palermo*. As in the previous case, this represents a trend on extreme events, more diffused in the East side of the island.
- Correlation metrics—C.*A* (Video_3): in this case a geographical clustering pattern was obtained, identifying eastern and western Sicily. This is coherent with the fact that the Correlation metrics finds shape similarities and it is less sensitive to the micro-climatic differences.
- Correlation metrics—C.*B* (Video_4): here the combination between dimensionality reduction and correlation metrics brings to a rough splitting of the island. The number of clusters does not exceed 3 and often very far away stations are grouped together in the same cluster.

The results of the 4 settings for the year 2021 are in line with our initial research hypothesis, for which the anomalies correspond to *extreme* stations. This behaviour was further confirmed by the Kruskal–Wallis test, as reported in the SI document.

Moreover, East Sicily emerges as the most *extreme* zone of the island, confirming the real occurred events reported in^47^ and discussed in^48^.

Finally, Video_2 shows that the use of weekly averaged data (C.*B*) gives rise to balanced presence of both anomalies and territorial clusters in the Euclidean case. On the other hand, Video_4 shows that C.*B*, differently from C.*A*, does not provide clear geographical splitting in the Correlation case.

In conclusion, in the annual case the use of Euclidean metrics led to detect the anomalies, while in contrast, the use of the Correlation metrics as a similarity measure allowed us to identify more uniform clusters.

#### Full clustering

We report here the full clustering results, obtained using C.*C* and C.*D* of Table 1. Similarly to the annual case, the use of Euclidean metrics brings to exceptions detection, by highlighting the presence of clusters composed by a unique site. We believe that the reason why anomalous clusters are independent lies on the fact that extreme events intensities are very different among sites^71^. Figure 3a shows the presence of one principal cluster and many anomalies, such as *Pedara*, *Augusta* and *Siracusa*. In contrast, Fig. 3b reports different principal clusters—geographically distributed—and only one exception: *Pedara*.

In order to validate results, we carried out a case by case analysis. First of all, the full and the annual clustering results in the case of Euclidean metrics (C.*A* and C.*C*, respectively) are compared in Fig. 3a by counting how many times the stations have been clustered as anomalous in the annual case. It turns out that the stations with an higher counter are the ones clustered as anomalies in the full case as well, except for *Catania*.

In any case, Fig. 3a confirms the results consistency, since East Sicily emerges as the most *extreme* side of the island.

The Kruskal–Wallis test was applied to the full case, providing similar results to the annual case. Among the characterizing indicators (“Clusters validation procedure” section), *md* (Maximum per day) and *h* (Heavy rain (%)) are particularly relevant in the full case.

Figure 3c,d show the *md* and the *h* heat-maps, in the full case. In contrast, Fig. 3a,b show the clustering results. Several similarities among the maximum values of the indicators and the anomalies can be observed. Therefore, also in the full case, the *extreme* stations coincide with the anomalous clusters. Moreover, the *red* cluster in Fig. 3b represents a cluster of *extreme* stations, confirmed by Fig. 3d. In fact, apart for the anomaly of *Pedara*, these stations retain the highest values of the *h* indicator.

**Figure 4:**
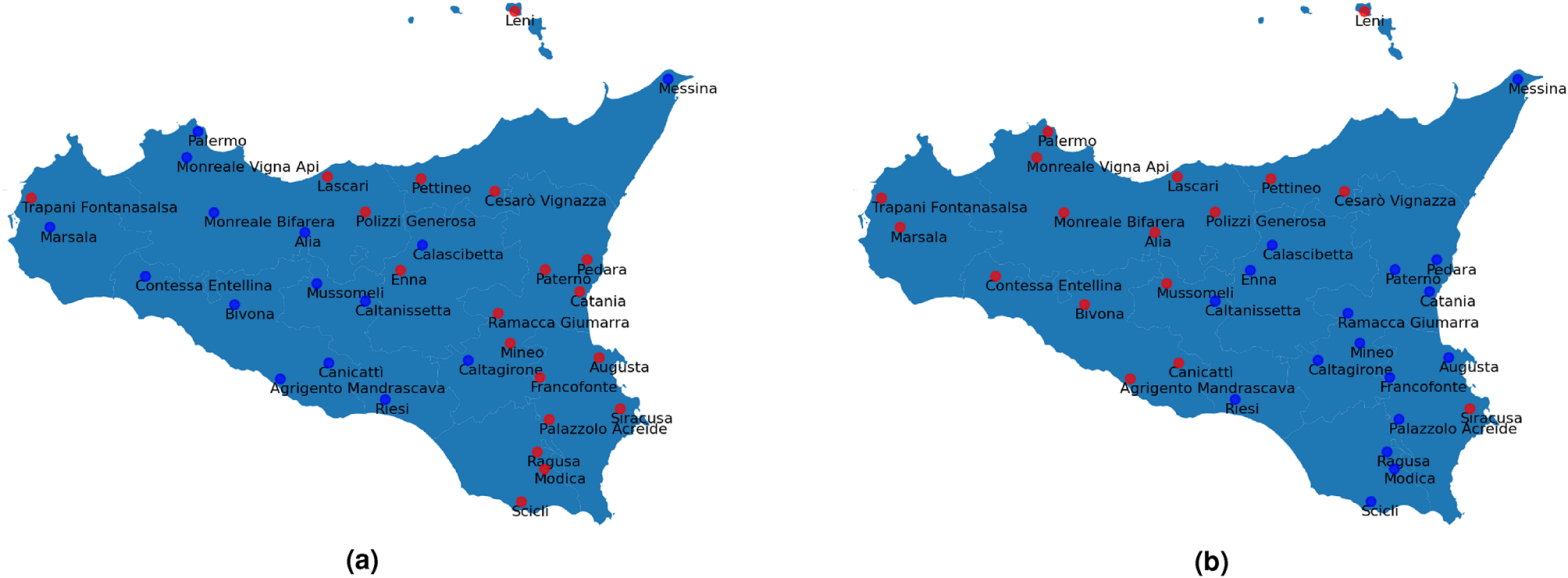
Full case—correlation metrics. (**a**) C.*C*. (**b**) C.*D*. The two clusters are reported in red and blue in both the panels.

In conclusion, the different implemented experimental settings allowed us to highlight several different aspects of extreme events. Certainly, the presence of these phenomena in eastern Sicily emerges both from the annual and the full clustering, especially when the Euclidean metrics is used as a similarity measure in the AP algorithm. On the other hand, the use of Correlation metrics brings to consider Sicily composed of two different climatic areas: West side and East side, as shown in Fig. 4, where there are only 2 large clusters. Moreover, in this case no similarities between characterizing indicators and clusters are found (compare Figs. 3c and 4a, Figs. 3d and 4b).

Eventually, the clustering involving C.*C* of Table 1 with the use of the Euclidean metrics seems to be the most performing setting—among those tested—in finding extreme events; whereas, collection C.*D* results as the most suitable arrangement to obtain geographically uniform clusters.

To confirm the latter results, firstly we compared our C.*D* findings with previous works in literature, obtaining similar geographical distributions^31,32,44^. In fact, in those works—as well as in Fig. 3b—there is a geographical splitting of the region highlighting the North, West, Center, East, and South-East Sicilian sub-regions.

Moreover, to further confirm the robustness of our findings, we compared our results with some physical indicators^71,72^. In particular, we evaluated the Kruskal–Wallis test between the clusters and the altitudes of the rain gauges for both collections C.*C* and C.*D*. In the case of collection C.*C*, the p value related to the *altitude* turns out not to be characterising for the clusters (p value = 0.31). Differently, in the case of C.*D* collection, the related p value is 0.03, confirming that the *altitude* characterizes the clusters. This suggests that the weekly mean data show a weak ability of representing extreme events, while they satisfactorily embed geographical aspects of the station gauges locations.

### K-means results

We further added a comparison between AP Clusters and K-Means. Using the C.*C* collection we find that, by setting the five AP case anomalies as initial centroids and varying the sixth centroid, we obtained three cases: in almost the 80% of experiments (22 over 29) the K-Means and AP Cluster perfectly coincide (Jaccard score = 1 for all of the clusters). Among the remaining seven experiments, four of them differ from the reference case by one or at most two stations in eastern Sicily, (Jaccard score $\le$ 0.5 at most one cluster; then, for only three experiments the difference is more significant (Jaccard score $\le$ 0.5 for at least two clusters).

**Figure 5:**
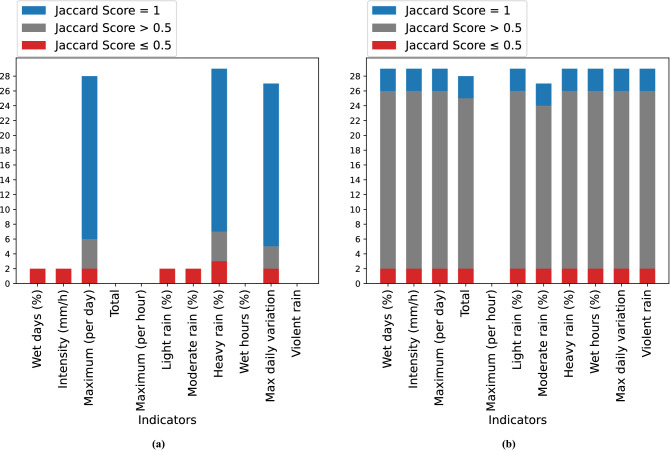
Characterizing indicators in the K-Means initial centroids-based experiments. The histograms count over the experiments how many times the indicators result as characterizing for the clusters (*p* value < 0.05), grouped by the categories defined by the Jaccard score. (**a**) C.*C* collection. Those experiments different from the AP case (in red) have many characterizing indicators, whereas those experiments similar or coinciding to the AP case (in blue and grey) have the same three characterizing indicator representing *extremeness*. (**b**) C.*D* collection. Almost every indicator is characterizing for each type of experiment.

For all the experiments, statistical validation was carried out with the Kruskal–Wallis test, as reported in Fig. 5a. In the first case, no difference in the p-values respect to the AP case is detected; in the second case, the characterising indicators coincide with the reference ones, i.e. *md* maximum (per day), *h* heavy rain (%) and *mv* max daily variation; in the third case, almost all the indicators are characterising and thus are not related to extreme events detection, differently from the AP results.

**Table 3:** 10 most frequent clusters (increasing order) obtained with K-Means clustering over the 200 runs.

Cluster	C.*C* Collection	C.*D* Collection
Cluster 1	Bivona, Contessa Entellina, Monreale Bifarera, Monreale Vigna Api, Palermo	Agrigento Mandrascava, Alia, Bivona, Calascibetta, Caltanissetta, Canicattí, Contessa Entellina, Enna, Marsala, Mussomeli, Riesi, Trapani Fontanasalsa
Cluster 2	Monreale Bifarera, Monreale Vigna Api, Palermo	Agrigento Mandrascava, Alia, Bivona, Calascibetta, Caltagirone, Caltanissetta, Canicattí, Contessa Entellina, Enna, Marsala, Mussomeli, Riesi, Trapani Fontanasalsa
Cluster 3	Monreale Vigna Api, Palermo	Leni, Messina
Cluster 4	Catania	Monreale Vigna Api
Cluster 5	Marsala, Trapani Fontanasalsa	Catania, Francofonte, Mineo, Paternó, Ramacca Giumarra
Cluster 6	Palazzolo Acreide	Modica, Palazzolo Acreide, Ragusa, Scicli
Cluster 7	Messina	Augusta, Catania, Siracusa
Cluster 8	Siracusa	Cesarò Vignazza, Lascari, Leni, Messina, Monreale Bifarera, Monreale Vigna Api, Palermo, Pettineo, Polizzi Generosa
Cluster 9	Augusta	Augusta, Siracusa
Cluster 10	Pedara	Pedara

The same analysis was done for the C.*D* collection of the weekly mean data. By fixing the centroids on the basis of the AP case, we get the same clustering. This was not as expected as in the C.*C* case, since this time we have more uniform clusters. Also in this case we did a sensitivity analysis with respect to the centroids of all clusters, finding that the 93% of experiments (27 out of 29) are coincident or similar (*JS = 1* and *JS > 0.5*) to our baseline result. The remaining two experiments ($JS \le 0.5$), however, turn out to be different. Unlike the C.*C* case, the statistical validity analysis showed homogeneous distributions among the characterizing indicators, as reported in Fig. 5b. In fact, even in our result, most of the indicators are found to be characterizing for the clusters. This confirms the goodness of our results on finding extreme events using the C.*C* collection with respect to collection C.*D*, where, as expected, the weekly averaging masks the presence of extreme events, obtaining a more uniform clusters distribution.

**Figure 6:**
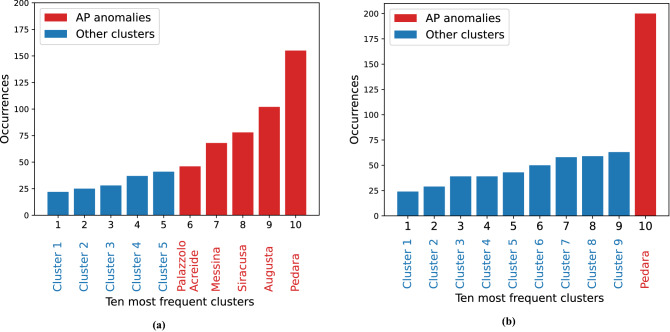
K-Means—clusters frequency analysis over 200 runs. (**a**) C.*C* Collection (High frequency data). The five most frequent clusters are the five anomalies of the AP case. The other five are clusters composed by different stations, as reported in Table 3. (**b**) C.*D* Collection (Weekly mean data). Except for *Pedara*, the most frequent clusters are different from the AP case. Those clusters are listed in Table 3.

Finally, we did an inter-cluster frequency analysis on 200 K-Means runs with random initial centroids for both C.*C* and C.*D* collections, reported in Fig. 6a,b, respectively. In the former case, we obtain that the most frequent clusters are precisely the five anomalies of the AP result. This confirms the robustness of our findings. In the second case, on the other hand, the only anomaly that is always present and corresponds to the most prevalent cluster, is Pedara, while for the remaining clusters there is not such a clear spread, as reported in Table 3.

### Local investigation

**Figure 7:**
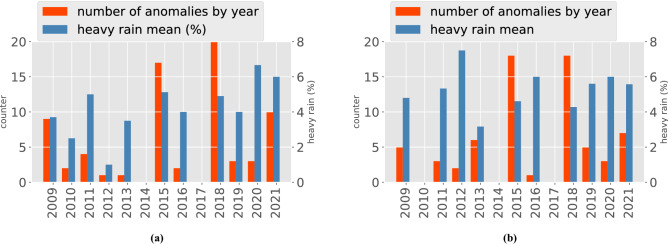
Anomalous years—Euclidean metrics. C.$A_{s}$ (**a**). C.$B_{s}$ (**b**). The heavy rain mean of the year *y* consists of the mean of the *heavy rain (%)* values for all the stations that cluster the year *y* as anomalous.

In the local case we investigated the temporal evolution of rainfall events. In particular, anomalous years in the entire observed period were detected. To this aim, the AP algorithm was applied only to C.$A_{s}$ and C.$B_{s}$, analysing one station per time. As in the geographical investigation, we chose to use both the Euclidean and the Correlation metrics. In order to understand the most *anomalous* years, we counted (over stations) how many times one year appears as exception when using the Euclidean distance. Figure 7a,b report the years counters in respectively C.$A_{s}$ and C.$B_{s}$ (in red).

In both cases the most anomalous years were 2015 and 2018. This means for instance that 2018 is clustered as anomalous in about 20 over 34 stations for Euclidean distance and C.$A_{s}$. We see that also the 2021 counter increases after the years 2019 and 2020. Additionally, Fig. 7a,b also report the *heavy rain (%)* mean values (in blue). In this case, we fix a year *y* and we compute the mean of the *heavy rain (%)* values for all the stations that cluster the year *y* as anomalous, thus obtaining, for instance, that 2020 and 2021 have the highest mean values. Summarizing, in the case of C.$A_{s}$ and Euclidean distance, an increasing trend on anomalous years was found concerning the heavy rain mean indicator (see Fig. 7a in blue). On the other hand, in C.$B_{s}$ and Euclidean distance, the trend is less detectable and the highest value of heavy rain mean is measured in 2012 (see Fig. 7b in blue).

## Conclusions

The main goal of this work was to introduce a clustering approach detecting extreme rainfall events occurred in Sicily, from 2009 to 2021 and to identify communities of sites with anomalous behaviors.

To the best of our knowledge, we are presenting for the first time in the literature, the use of multi-modal clustering analysis to detect extreme rainfall events in Sicily. With this approach, we were able to confirm and expand some preliminary observations presented in^32^, where a statistical approach has been applied to analyse rainfall trends. Specifically, in our work a clustering technique, the Affinity Propagation algorithm, was employed to confirm and discover geographical and historical rainfall patterns.

In order to understand the rainfall phenomena mostly characterizing the geographical communities identified, several rainfall indicators were introduced^69^ and evaluated over the available time series. In addition, the obtained results were validated by means of the Kruskal–Wallis statistical test.

Three types of clustering analysis were conducted—full, annual and local: firstly applied to the entire high frequency time series and then applied to the weekly averaged data. The reason of this choice lied in the need of reducing the dataset size in order to verify the robustness of the results. Eventually, we investigated both the Euclidean and the Correlation metrics as distance measures for the AP algorithm.

The paper presents several significant findings:

- East Sicily is increasingly becoming a protagonist of *extreme* events, both in the full period of recordings and in the annual cases, confirming the^32^ findings. This result is more evident choosing the Euclidean metrics in the implementation of the Affinity Propagation algorithm.
- High frequency data (C.*A*) with the Euclidean metrics brings to the detection of an increasing trend over years of *extreme events*; in contrast, C.*B* of weekly averaged data does not provide the same evidence.
- 2021 emerges as one of the most anomalous years in the local investigation over time. Moreover, we found from the geographical analysis -reported in the SI document- that it is characterized by *extreme* events in the East side of the island, particularly in the cities of *Catania*, *Siracusa* and *Augusta*.
- Using a statistical validation approach, we found out that three indicators describe the anomalous clusters finely: the maximum per day (*md*), the maximum daily variation (*mv*) and the heavy rain percentage (*h*). This entails that most of the time anomalous clusters are characterized by the presence of *extreme* events. The statistical significance of these indicators is confirmed by the sensitivity analysis performed with the K-Means algorithm.
- The Affinity Propagation algorithm allowed to detect anomalies, namely *extreme* stations, considering the full dataset. Specifically, using the Euclidean metrics, the cities of *Augusta*, *Siracusa*, and *Pedara* were identified clearly as anomalous at the first iteration of the algorithm. In contrast, *Palazzolo Acreide* and *Messina* have been detected at the second and third run of the algorithm, respectively. *Catania* does not emerge as an anomalous cluster, however, similarly to the previous sites, it presents 6 out of 13 anomalous years. These results were confirmed through statistical analysis and in-depth comparison with the K-Means algorithm.
- The Euclidean metrics is sensitive to micro-climatic changes, i.e. geographically close stations are clustered in different groups. This is consistent with what can be experimentally observed since there are actually rainfall events of different frequency and magnitude a few kilometers apart. Moreover, the geographical properties of the clusters have been confirmed by using orographic indicators such as the altitudes of the gauges stations.
- The Correlation metrics allowed to identify uniform clusters. This is particularly evident in the full case, in which the algorithm splits Sicily in East and West parts.
- High frequency data (C.*C*) and Euclidean metrics emerge as the most suitable setting to find extreme rainfall events in the geographical clustering. The dataset size reduction by weekly means (C.*D*) is successful to find geographically uniform clusters. In particular, it merges together anomalies and territorial clusters in a balanced way, finding also appropriately *extreme* clusters, such as the one in eastern Sicily.

We are aware that the obtained results are no more robust than in previous before-mentioned studies, given the short observational period and therefore potentially affected by the large unforced internal/natural variability. It is clear that a wider spatial and temporal range would be needed to fully validate changes in heavy rainfall trends^73–76^. However, this paper has a methodological focus aimed to introduce an elegant approach to detect extreme events. In fact, the clustering approach is promising to interpret spatial patterns of heavy rainfall.

Further research is necessary to determine which dimensionality reduction procedure is the best for having a more precise local investigation, which could take into account both the extreme events detection and the geographical and environmental distribution of the station gauges. For instance, the *total per hour* datasets, the *daily averaged* datasets or some features refinement techniques, as the Principal Component Analysis, can be advantageous to reduce the features-set dimensionality. More accurate rainfall indicators could be applied and derived, in order to entirely characterize rainfall extreme events. Additionally, in order to have more robust results, a general analysis can be conducted, merging stations by provinces, considering a temporal clustering over the entire region, or even increasing the temporal range of investigation. In this way, the methodology introduced may potentially also yield robust findings in terms of a climate change signal.

Moreover this study could contribute significantly to the development of the decision support systems based on multi-modal and easy to use data science tools for policy makers, stakeholders, and social actors. In general, there are many concerns about the social and economic consequences of climate change; the more vulnerable local territories are, the greater the worries and the possible harms. Sicily is subject of numerous studies in this context (see^77,78^). The main climatic events covered in this context are the rising of sea levels, extreme sea and air temperatures, and drier conditions^77^. Our work lies within this framework, since the clustering method finds the extreme rainfall events in a particular territorial area (in our case eastern Sicily). Therefore, the article allows local governments to become more aware of the aforementioned territorial criticality, and, consequently, it is possible to make targeted and non-dispersing prevention investments. For instance, one of the management objectives is about prevention. Policy makers could think of installing traffic lights on roads with flood warnings for the population, majorly in areas resulting as extreme. Another example of a policy could concern education: in the most extreme areas, evacuation/extreme event management tests could be frequently scheduled in schools or offices to provide knowledge and skills for the emergency management.

This study contributes significantly to disseminate knowledge and awareness of extreme events, in the framework of public engagement with citizens. Rather than showing pictures of melted glaciers or giving the single news story of a flood, we have implemented a method that analyses and fuses multi-modal datasets, in an understandable way. In fact, relatively little scientific methodological knowledge is needed to understand the results. This could therefore be a way of bringing the scientific findings closer to the population.

This work is also useful in bringing the topic of climate change back to a dimension of data analysis, helping to produce greater awareness in the population and thus triggering virtuous behaviours in citizens, stakeholders, organizations and political institutions. Future studies, that we would like to pursue, concern precisely the dynamics necessary to achieve a reverse social tipping point, which can lead sustainable actions to become social norms. We believe that such kind of works could be crucial to trigger a social change. We are currently working on the possible development of a demo, based on the proposed method, to be later distributed to potential investors and researchers in the field.

## Supplementary Information

Supplementary Information 1.

Supplementary Video 1.

Supplementary Video 2.

Supplementary Video 3.

Supplementary Video 4.

## Data Availability

The dataset together with the code is available at the following GitHub Repository^54^.
