# Supplementary material for: A multi-modal machine learning approach to detect extreme rainfall events in Sicily
Source: Sci Rep. 2023 Apr 16;13:6196. doi: 10.1038/s41598-023-33160-9 (PMC10106478; doi:10.1038/s41598-023-33160-9)
Supplement: Supplementary file 1 — Supplementary Information 1. [file 41598_2023_33160_MOESM1_ESM.pdf]

Supporting Information for

A Multi-Modal Machine Learning Approach to Detect Extreme Rainfall Events in Sicily

Eleonora Vitanza, Giovanna Maria Dimitri, Chiara Mocenni

Chiara Mocenni
E-mail: chiara.mocenni@unisi.it

This PDF file includes:

Supporting text
Figs. S1 to S23

Supporting Information Text

Annual variables analysis

In this section the annual histograms will be displayed, in order to better understand which stations are more affected by extreme events. Figure S1 shows some of those histograms, in particular the ones relative to *Catania*, *Palermo*, *Messina*, *Trapani Fontanasalsa*, *Siracusa* and *Palazzolo Acreide*.

Figure S1g shows an increasing trend in Catania for the *dmax* variable, with a peak of about 160 *mm* in 2020. At the same time, we can appreciate in figure S1m a lower *rd* annual value. Same behaviour can be seen in *Palermo* for the same year (figure S1h and S1n).

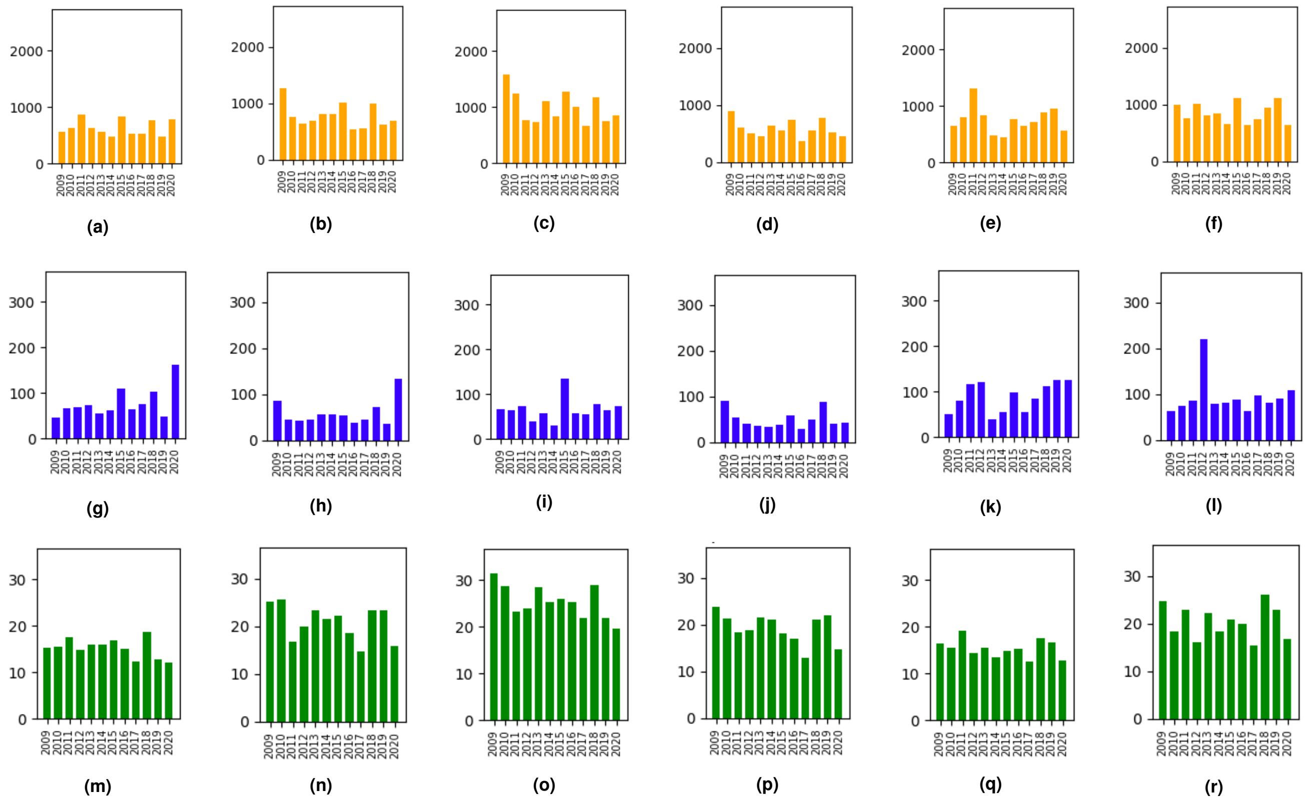

Fig. S1. Annual variables of *Catania* (first column), *Palermo* (second column), *Messina* (third column), *Trapani Fontanasalsa* (fourth column), *Siracusa* (fifth column) and *Palazzolo Acreide* (sixth column). Orange histograms represent the *tot* annual variable; blue histograms represent the *dmax* annual variable; green histograms represent the *rd* annual variable. The definition of those variables is reported in the main document. In the x axis of every histogram there are the years between 2009 and 2020.

Since this happened in 2020, and not other years, this observation led us to hypothesize the presence of an increasing in extreme rainfall events, in terms of frequency and intensities. This was the rationale we used in our work to select the stations, counting how many years have this property in a station. Moreover, in *Palermo* we could appreciate a decreasing trend of the *tot* variable (Figure S1b).

This means that an increasing in the maximum values comes with a decreasing in the total annual rainfall. This in some way seemed to confirm the observations made in the city of *Palermo*.

Figure S1 shows different behaviours for the cities of *Messina* and *Trapani Fontanasalsa*. In *Messina* the *dmax* peak lies in 2015, which is associated with high *rd* and *tot* values. Despite such high values, the *dmax* value of about 135 *mm* is not negligible (figure S1i). In fact, it represents an extreme year for what concerns the presence of rainfall events.

In 2020, in contrast, there was the lowest local *rd* value, associated with a low *tot* value and a medium/high *dmax* (figures S1c, S1i and S1o). This suggests a global reduction of rainfall events in that year but with a considerable maximum per day. Again this case represents the typical extreme rainfall events setting.

In *Trapani Fontanasalsa* we see maximum peaks in 2009 and 2018 (figure S1j), together with high values on total rainfall and rainy days in those years (figures S1d and S1p). Moreover we can see a notable local trend both in *Messina* and *Trapani Fontanasalsa* for a decreasing on the total annual amount of rain and on the percentage of rainy days.

Finally, figure S1 shows also *Siracusa* and *Palazzolo Acreide* annual data. In *Siracusa* we have maximum per day peaks of about 100 *mm* (figure S1k). For instance, 2019 and 2020 share a similar *dmax* value, but they represent two different cases. In 2019 we have high *rd* and *tot* values, whereas in 2020 we have low values for them, which means again presence of extreme events (figures S1e and S1q). A similar reasoning could be done for 2012 and 2013. In *Palazzolo Acreide* we have a huge

dmax peak of more than 200 *mm* during 2012, associated with low values of the other variables (figures S1f, S1l and S1r). We observed the same for 2020, even if with a much lower peak.

1. Data visualization

We report here an increasingly zoom of *Augusta* rainfall time series, with the aim of better understanding rainfall behaviour over time. *Augusta* is located in the province of *Siracusa*, in the South-East of Sicily. We considered it as an example, but we could do the same for any rain gauge.

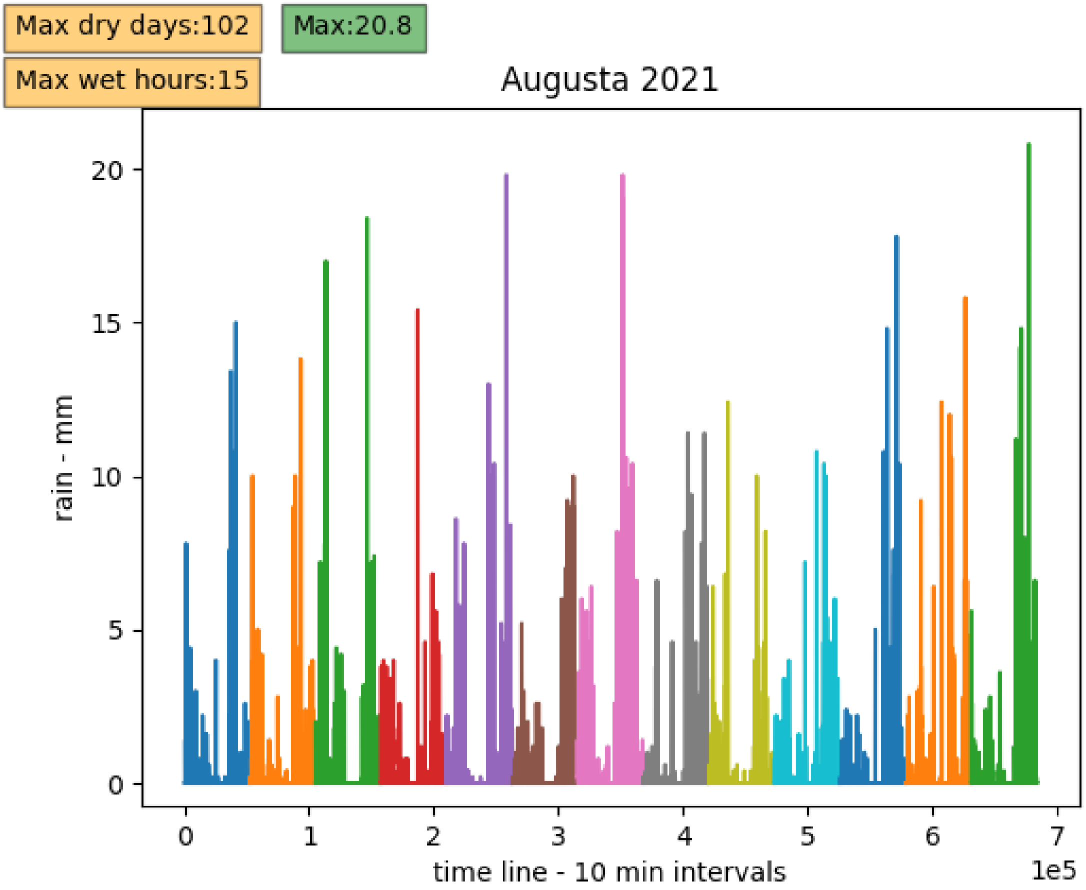

Fig. S2. Full view - Augusta

A. Full view. Figure S2 shows all the available data of *Augusta* over time. In the x axis there are all the 10 minutes steps from 01/01/2009 at 00:00 to 01/01/2022 at 00:00, while in the y axis there are the amounts of rain - in *mm* - for each temporal step. Colors allow to distinguish between different annual records collections.

. We consider three variables in the top-left of the figure:

1. *Max dry days*: it counts how many days of dryness there are. In other words, the longest dry period from 2009 to 2021 in *Augusta* consists of 102 consecutive days.

2. *Max wet hours*: it counts how many hours of wetness there are. In other words, the longest wet period from 2009 to 2021 in *Augusta* lasts 15 consecutive hours.

3. *Max*: it counts the maximum amount of rain in 10 minutes over the entire time series.

The *Max* variable indicates 20.8 *mm* of rain in 10 minutes. The plot shows clearly that this maximum value lies in the end of 2021. This is a confirmation of what we actually observed before choosing this study topic. Nevertheless, we observed a general trend over years to have a peak at about 20 *mm*. Namely, 2011 (the first in green), 2013 (in purple) and 2015 (in pink) have a similar peak to 2021.

Therefore, globally, we cannot think of a general increasing trend in peaks. In fact, we also notice a reduction in 2016, 2017 and 2018. Another observation is that all of those peaks lies, as expected, in the rainy season, that is in the last part of the years.

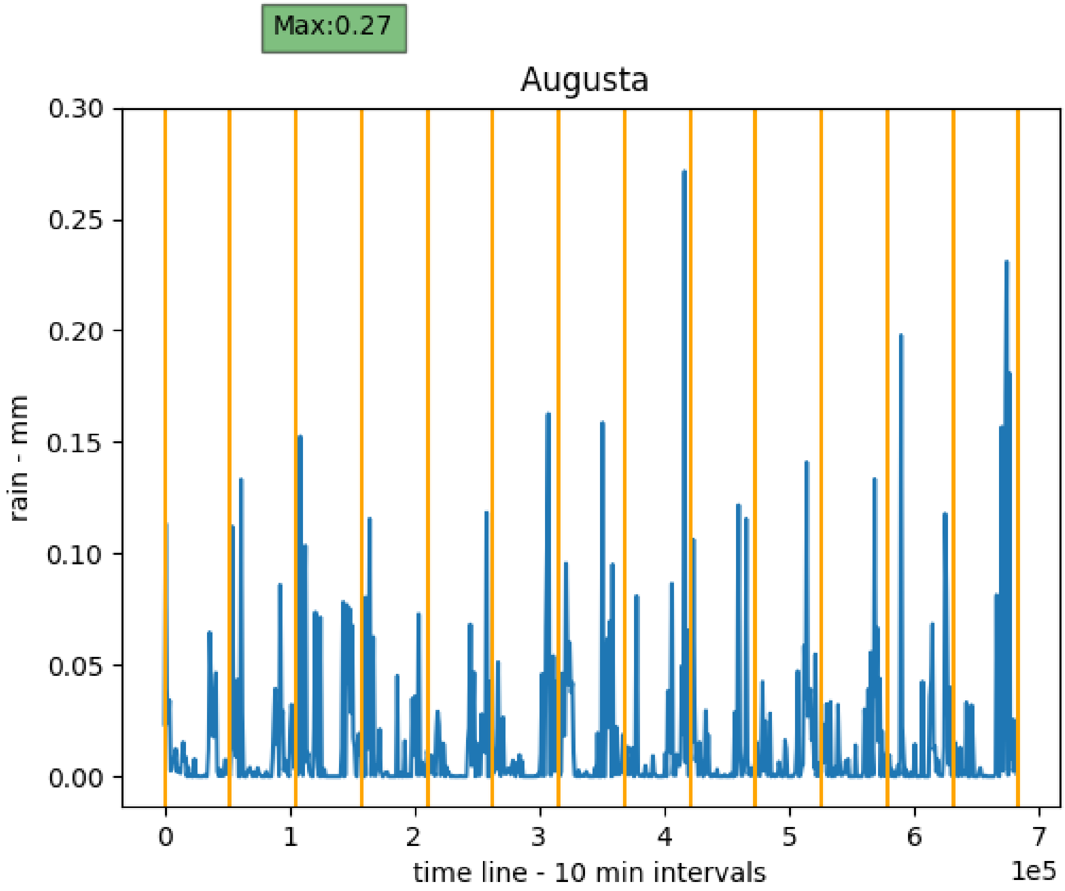

Fig. S3. Weekly mean - full view - Augusta

Moreover, the *Max dry days* variable lies in 2011, while the *Max wet hours* one lies in 2012.

Therefore, at first glance there does not seem to be an increasing dryness associated with a decreasing wetness trend in Augusta. However, considering only the extreme consecutive periods is not sufficient to claim that. In confirmation of this, extreme events main characteristic is represented by water bombs.

Consequently, we could have an increasing of dryness periods without continuity, observing short-duration rainfall events among a general drought. The idea is that those short-duration rainfall events have actually more and more higher intensity and frequency.

During all these observations, we also look at *weekly mean data*. We plotted only one value per week, which is the mean of the 10 minutes intervals. Figure S3 does not seem to lose relevant information respect to figure S2, except for the maximum mean value founded in 2016. Besides, this case deals with far fewer data and avoids noise. This suggests the idea developed in the main document to use mean data instead of the original ones, in order to reduce computation complexity during the run of the algorithms and to reduce outliers. We computed for every week the mean over the 1008 weekly data. The resulting dataset composed by 679 values is then reported in the figure S3.

It is particularly evident the difference between 2009 and 2021 mean values. In fact, the max mean value of 0.27 *mm* relative to the 2016 is the only value higher than the max 2021 mean value, while the max mean value in 2009 is of about 0.06 *mm*.

Moreover, in general an increasing trend on peaks over years is quite visible, except for the peak in 2016. Actually, the trend is more noticeable than in figure S2, thanks to the reduction of noise. This suggested that the averaged dataset could lead to interesting conclusions.

B. Annual view. The following figures show the annual data from 2009 to 2021. They represent 13 different zooms of the full plot. The structure of the figures is the same as before, but this time colors represent different months.

In 2009 we observed the *Max dry days* value of 39 achieved between July and August, and the *Max wet hours* value of 8 achieved in January. From this graphic we see seasonality, in fact the higher peaks lies in the autumn season, which is considered the rainy season for the Mediterranean area. There are some anomalies in November, due to the absence of peaks.

Moreover, we observed a very similar situation in 2010, this is why we do not report its plot. In this case the *Max dry days* value of 36, achieved between July and August, is lower than the one in 2009 and the *Max wet hours* value of 11 achieved in March is higher than the one of 2009.

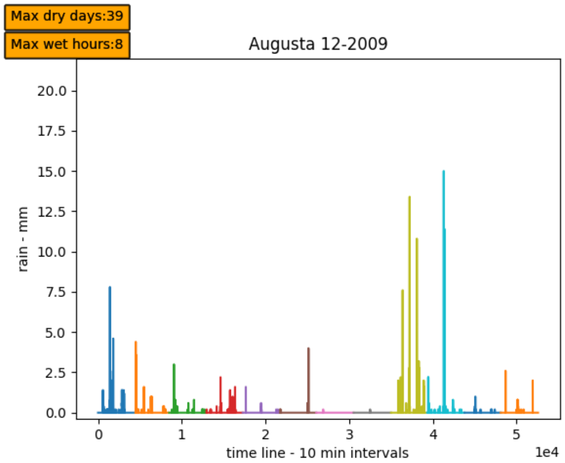

(a) Annual view - 2009 Augusta

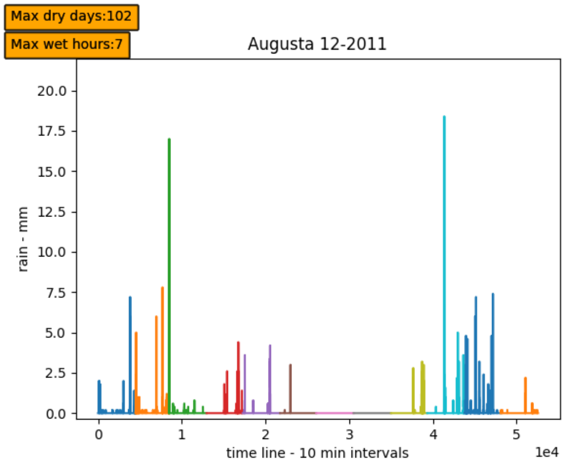

(b) Annual view - 2011 Augusta

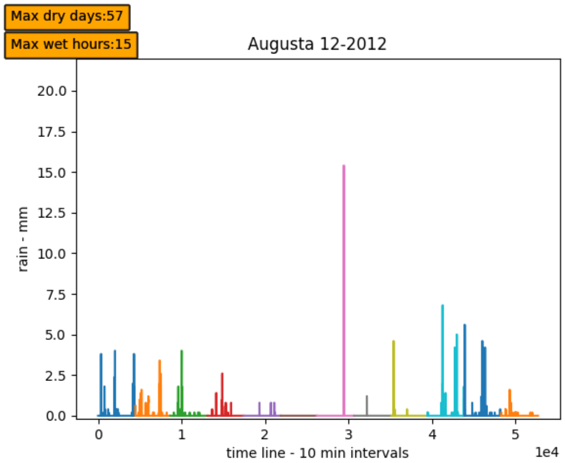

(c) Annual view - 2012 Augusta

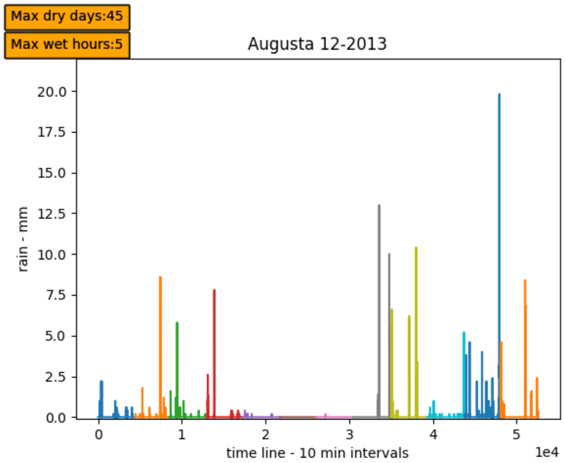

(d) Annual view - 2013 Augusta

Fig. S4. Annual view - Augusta

2011 is very different from the other years. Its *Max dry days* value coincides with the global one, therefore it is the driest year. This behaviour is also associated with rainfall peaks higher than 2009 and 2010 in the rainy season.

On the other hand, 2012 represents the wettest year - in the sense of continuous rain - since its *Max wet hours* value achieved in February coincides with the global one. Moreover, 2012 reached the maximum value in July. This behaviour is anomalous and shows the increase of the rainstorms phenomenon.

Moreover 2013 shows more or less a standard behaviour for Sicily, with dryness in the warmer months and wetness in the colder ones. We actually observe some anomalous peaks in August which refer to extreme unexpected rainfall events and a high annual peak of about 20 *mm*.

Furthermore 2014 did not highlight any remarkable event. This is why its plot is not reported here.

2015 has its peak in September, achieving again about 20 *mm*. It is a very high value for a 10 minutes temporal step, in fact 2016, 2017 and 2018 never achieve that.

2017 unusually has its peak in April, whereas the rest of its behaviour seem standard. 2015, 2016 and 2018 show again peaks of about 10 *mm* in the summer months. 2019 shows a standard evolution, again with some peaks in July. For this reason we decided not to report it here.

Eventually, 2020 showed an unusual October with no peaks, whereas 2021 reveals a tendency of very low rain during the year, except for September, October and November, where in contrast the peak coincide with the global one. This indicates a sort of redistribution of rainfall events over the years.

The general trend passed from an annual balanced distribution, for example in 2011 or 2013, to an unequal one. In other words, from this annual observation we conclude that in Augusta it rains less frequently than before but harder and especially in unexpected periods.

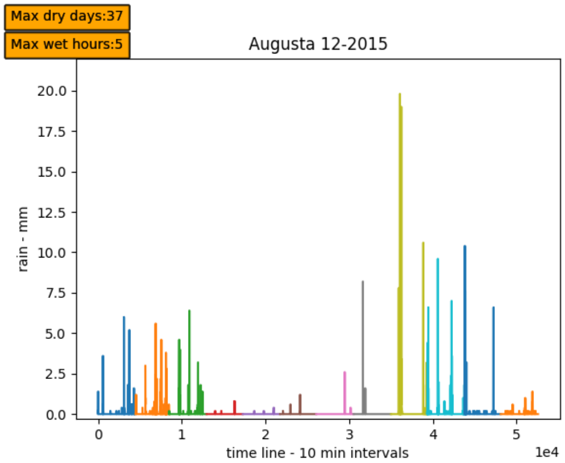

(a) Annual view - 2015 Augusta

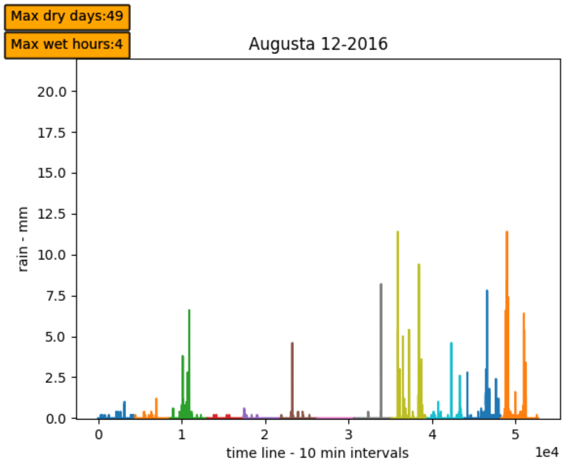

(b) Annual view - 2016 Augusta

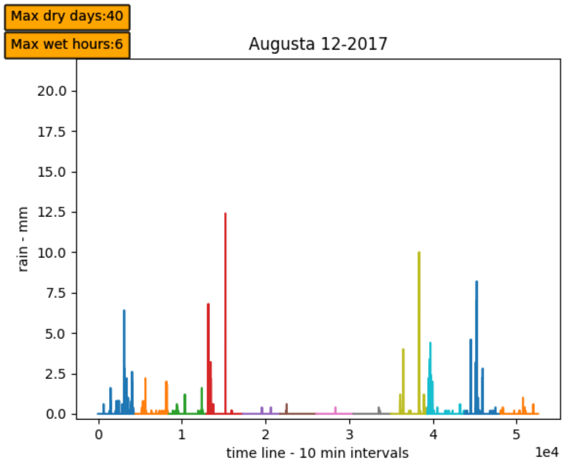

(c) Annual view - 2017 Augusta

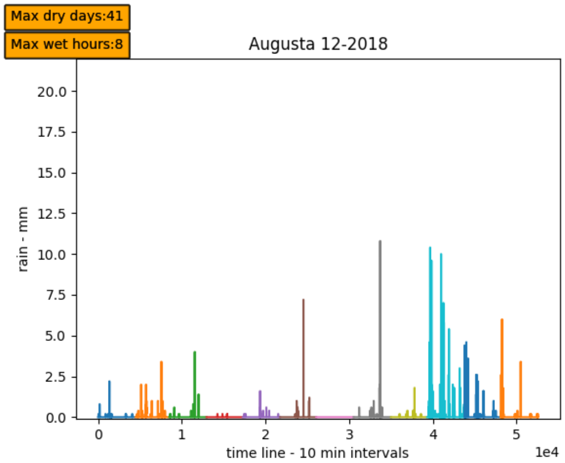

(d) Annual view - 2018 Augusta

Fig. S5. Annual view - Augusta

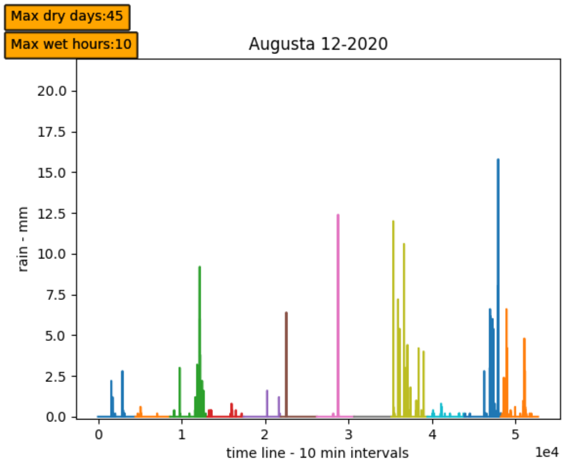

(a) Annual view - 2020 Augusta

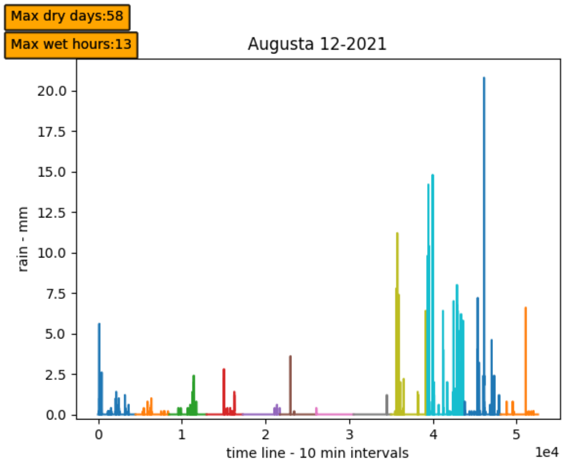

(b) Annual view - 2021 Augusta

Fig. S6. Annual view - Augusta

C. Monthly view. We report here the November distributions over years. We take it as an example, but we could do the same for any month, obtaining different new observations. Figure S7 shows November in the first 6 years of our investigation, from

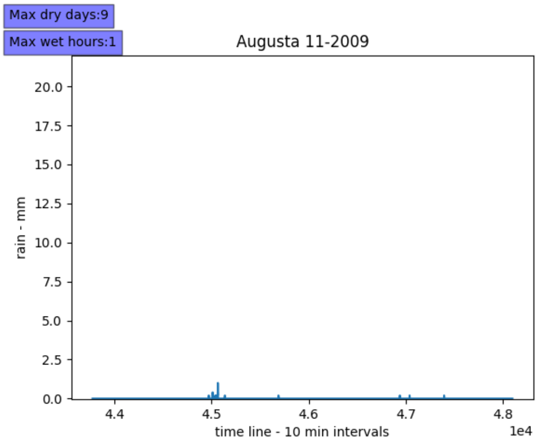

(a) 2009

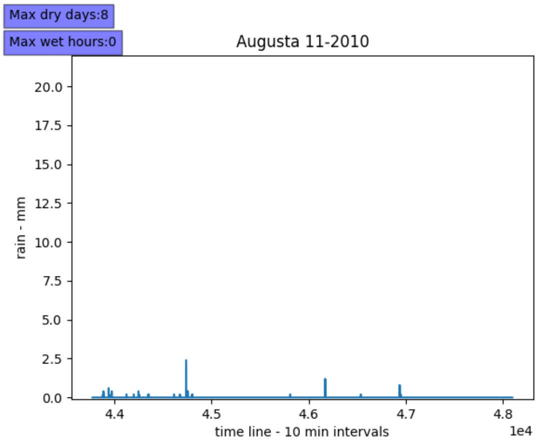

(b) 2010

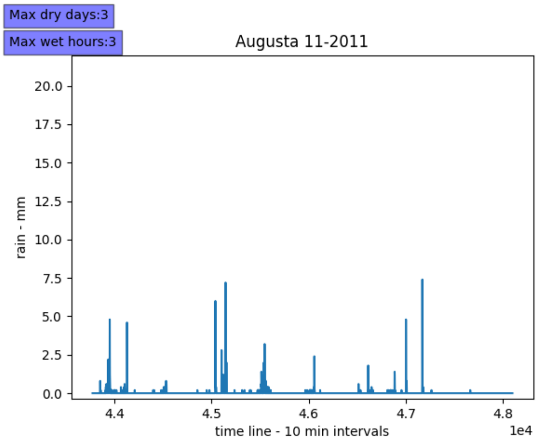

(c) 2011

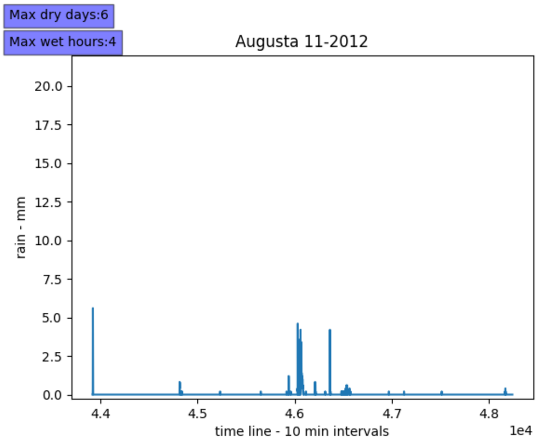

(d) 2012

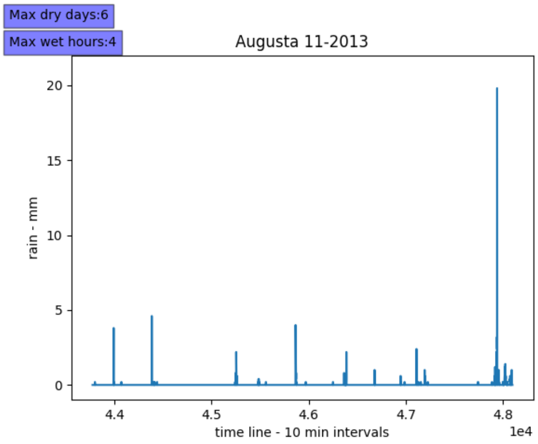

(e) 2013

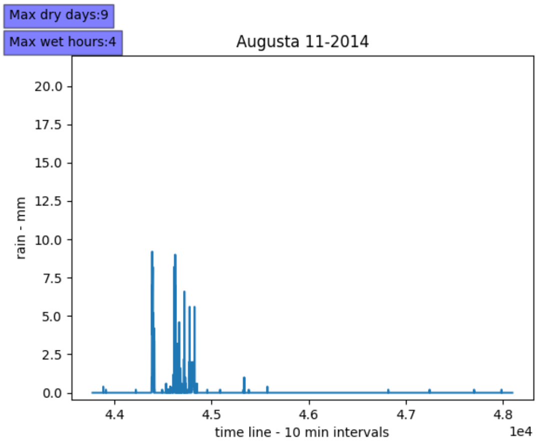

(f) 2014

Fig. S7. Monthly view - November - Augusta

2009 to 2014. We see that November rainfall events are very rare in 2009 and 2010, whereas they are common in 2011. The other three years show several rainfall peaks, notably the one of about 20 mm in 2013.

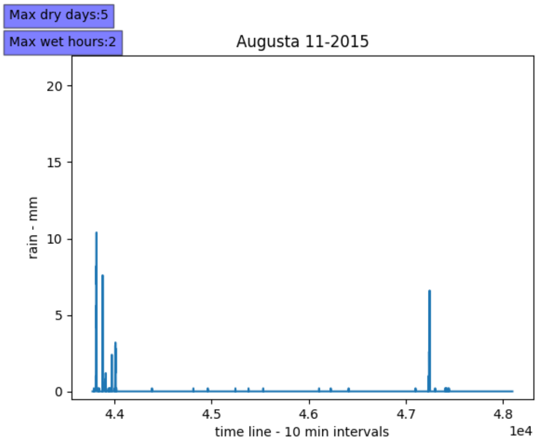

(a) 2015

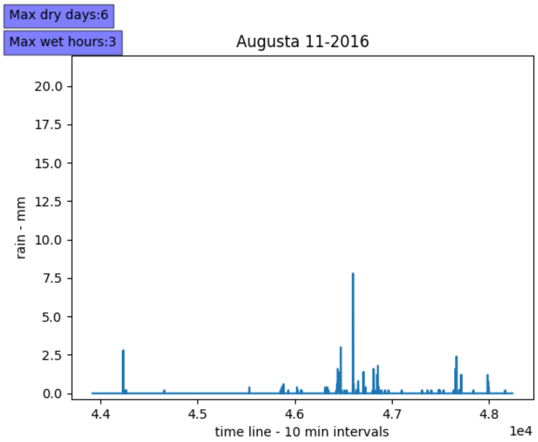

(b) 2016

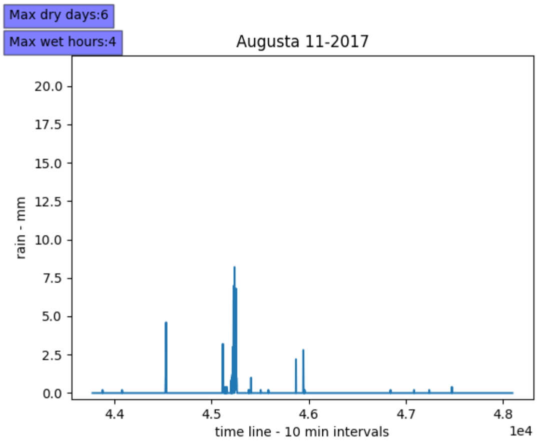

(c) 2017

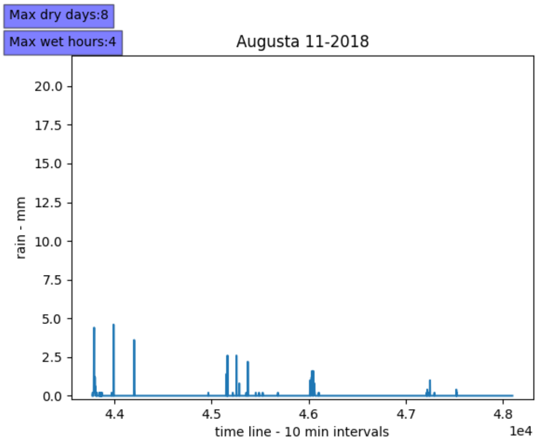

(d) 2018

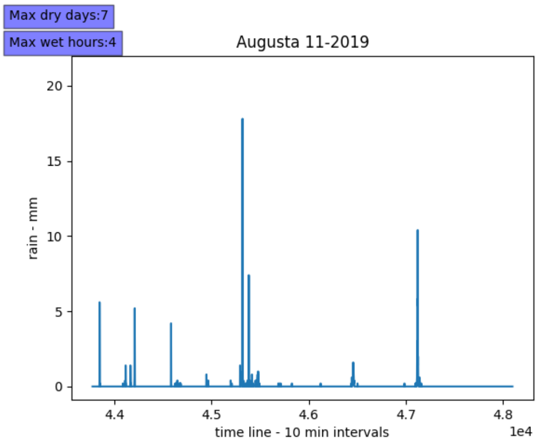

(e) 2019

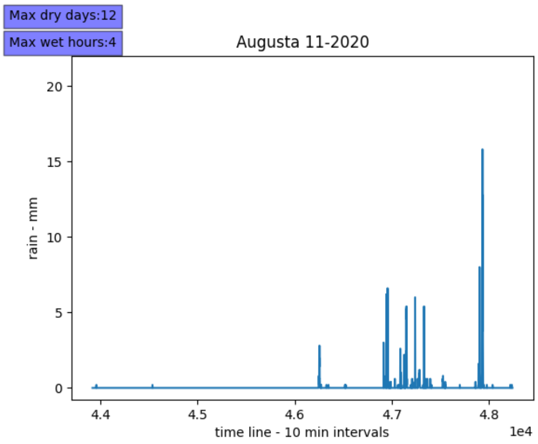

(f) 2020

Fig. S8. Monthly view - November - Augusta

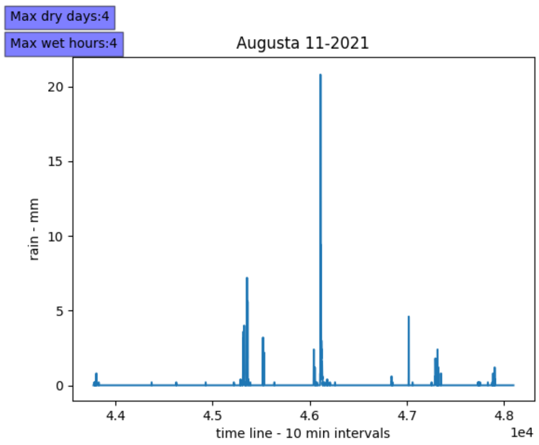

(a) 2021

Fig. S9. Monthly view - November - Augusta

Moreover figures S8 and S9 show November in the last 7 years of our investigation, from 2015 to 2021. Again we observe a general trend to storms with peaks reaching more or less the global maximum of Augusta, for instance in 2019 and in 2021. This is an alarming observation that confirms a change in the climate of Augusta. Data suggest that those huge peaks will be more and more frequent, becoming a threat to the environment and the population.

2. Annual results

Here we report the annual clustering results in all the considered settings. In particular, in the case of Euclidean metrics and C.A (see the main document for the Collections descriptions), the results consist in a principal cluster and some exceptions, mainly in the East side of the island. This suggests the vulnerability to extreme events of the East side of Sicily respect to the West. In the case of C.B, there are many principal clusters and some exceptions, again mostly in the East side. Differently, in the cases of Correlation metrics with both C.A and C.B, most of the time results consist in two clusters splitting Sicily in half. It follows that Euclidean metrics let to better detect outliers respect to the Correlation metrics. Consequently, the Euclidean metrics seems to be more suitable and precise than the Correlation metrics.

Figure S22 shows the exceptional rainfall events occurred in East Sicily in 2021. In fact, among the characterizing indicators obtained with the procedure explained in the main document, md (maximum per day) is found to be particularly relevant. Panel S22a shows the presence of a principal cluster and some anomalies, for instance *Catania*, *Augusta* and *Siracusa*. Differently, panel S22b presents several principal clusters distributed in the North, in the center, in the North-East, in the South-East and in the eastern center; in this case only *Augusta* and *Siracusa* are clustered as anomalous by the algorithm. Panels S22c and S22d represent the md indicator geographically referenced and value-based, respectively. Comparing independently panels S22a and S22b, with panel S22c, we observe a coincidence between the maximum values and the anomalies in the clusters. In particular, most of the anomalies in panel S22a represent the highest values of the md indicator in panel S22c or in panel S22d. The same happens with panels S22b for the two anomalies of *Augusta* and *Siracusa* and for the locations in the *light blue* cluster in panel S22b, which show the second highest values of the md indicator, with the only exception of *Contessa Entellina*. The same statement, not reported here, has been found also for the mv (maximum daily variation) indicator. Therefore, in 2021 the anomalous clusters consist of the stations with the highest md values. Moreover, East Sicily emerges as the most *extreme* zone of the island.

On the other hand Figure S23 shows that using the Correlation metrics, no coincidences between characterizing indicators and clusters are found for the year 2021. Actually, this happens in all of the other annual cases and in the full cases as well, in agreement with the fact that Correlation metric is less sensitive to outliers than the Euclidean one.

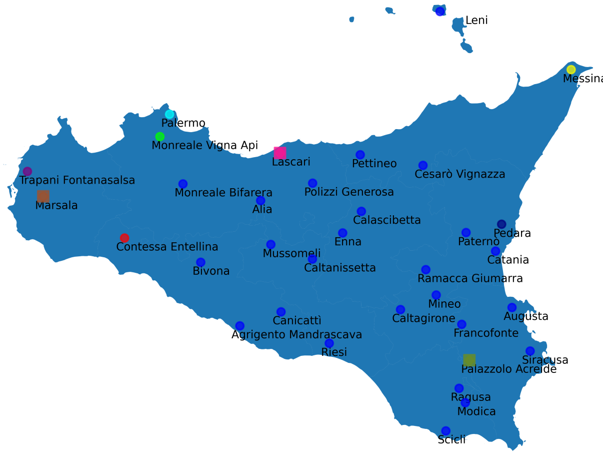

(a) Euclidean metrics and C.A

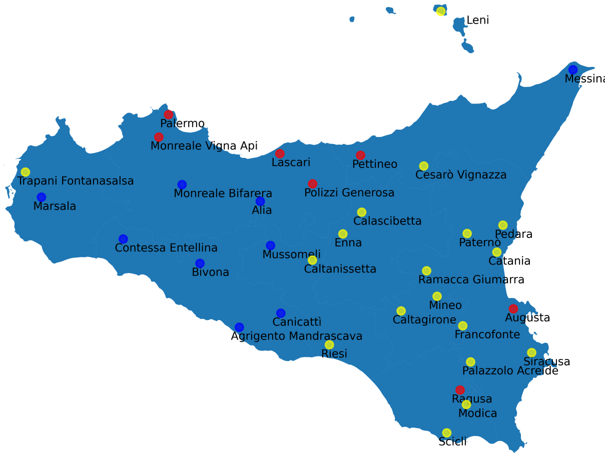

(b) Correlation metrics and C.A

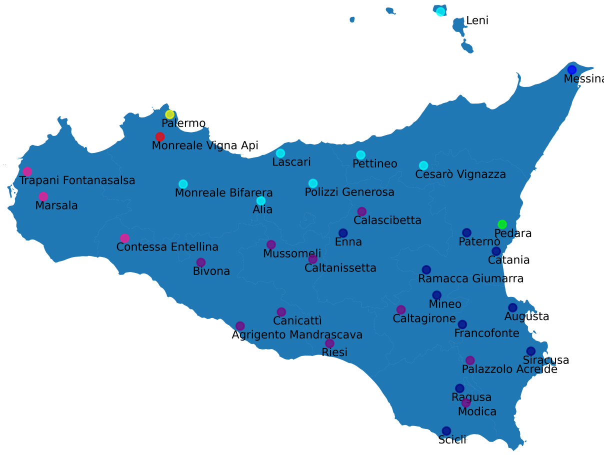

(c) Euclidean metrics and C.B

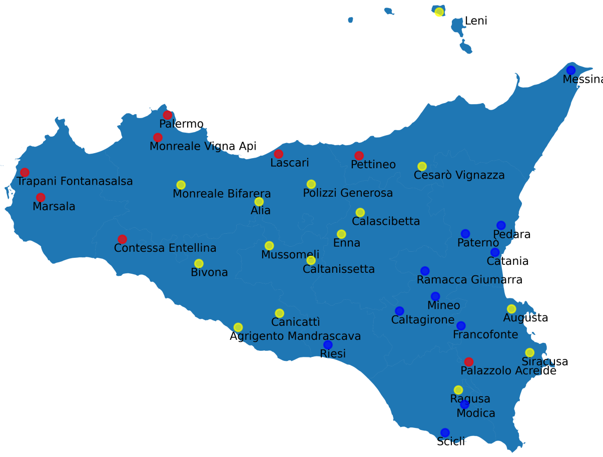

(d) Correlation metrics and C.B

Fig. S10. Annual case - 2009
Panel a: The principal cluster is reported in blue. **Panel b**: The three clusters are reported in blue, red and yellow. **Panel c**: The four principal clusters are reported in light blue, dark blue, purple and pink. **Panel d**: The three clusters are reported in blue, red and yellow.

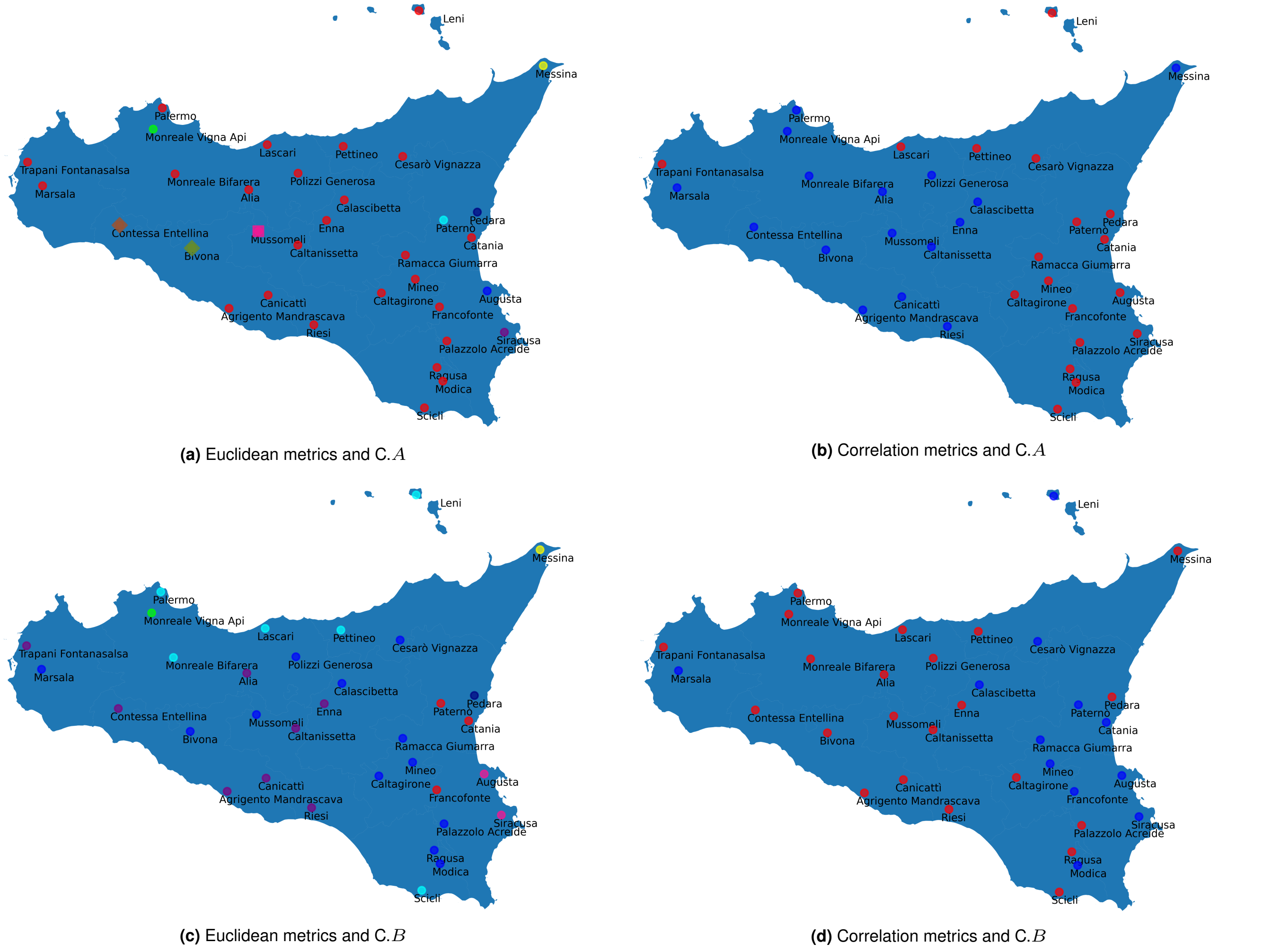

(a) Euclidean metrics and C.A

(b) Correlation metrics and C.A

(c) Euclidean metrics and C.B

(d) Correlation metrics and C.B

Fig. S11. Annual case - 2010

Panel a: The principal cluster is reported in red. **Panel b**: The two clusters are reported in red and blue. **Panel c**: The five principal clusters are reported in blue, red, light blue, pink and purple. **Panel d**: The two clusters are reported in red and blue.

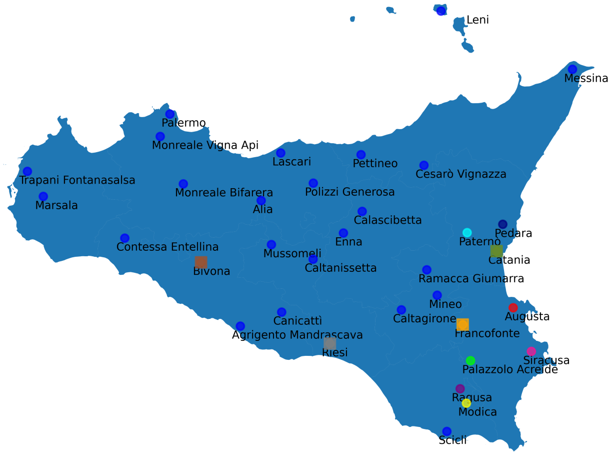

(a) Euclidean metrics and C.A

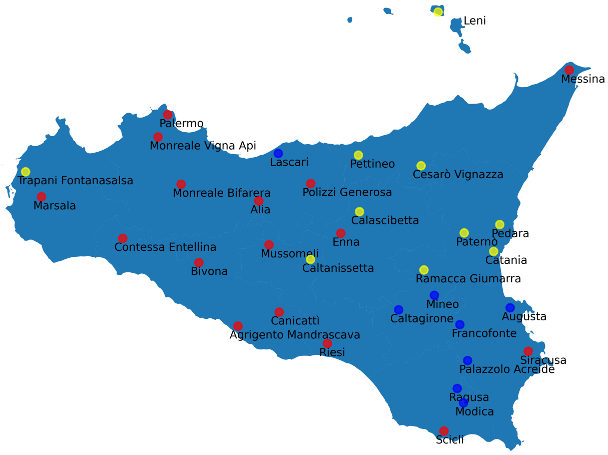

(b) Correlation metrics and C.A

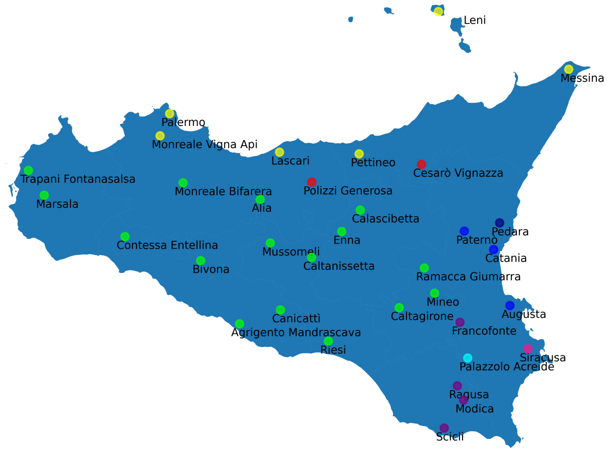

(c) Euclidean metrics and C.B

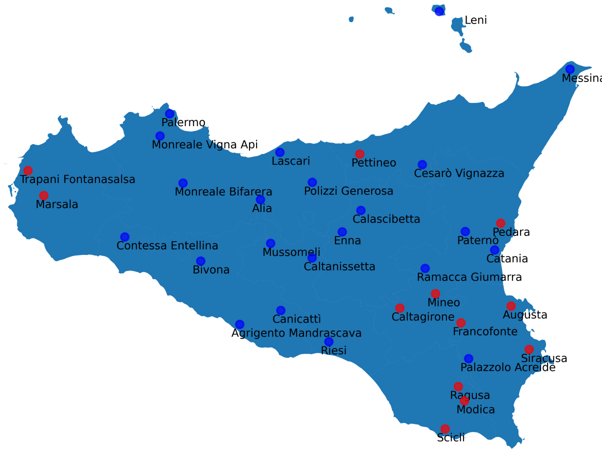

(d) Correlation metrics and C.B

Fig. S12. Annual case - 2011

Panel a: The principal cluster is reported in blue. **Panel b**: The three clusters are reported in red, blue and yellow. **Panel c**: The five principal clusters are reported in green, purple, blue, red and yellow. **Panel d**: The two clusters are reported in red and blue.

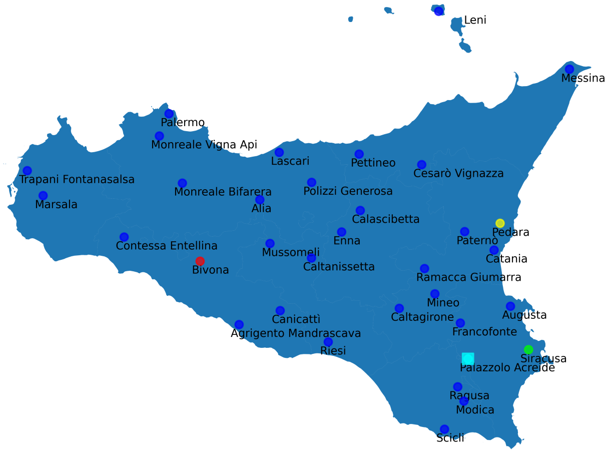

(a) Euclidean metrics and C.A

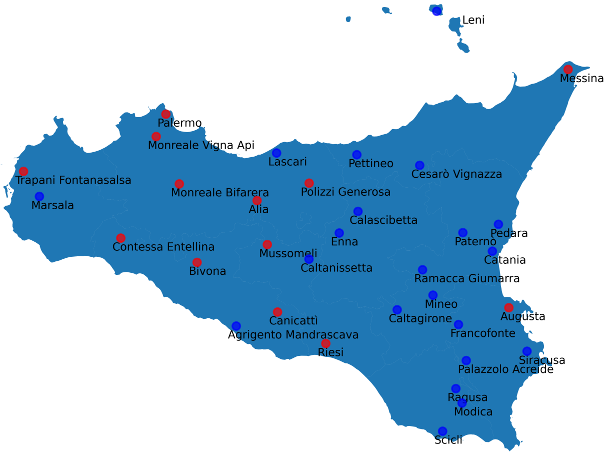

(b) Correlation metrics and C.A

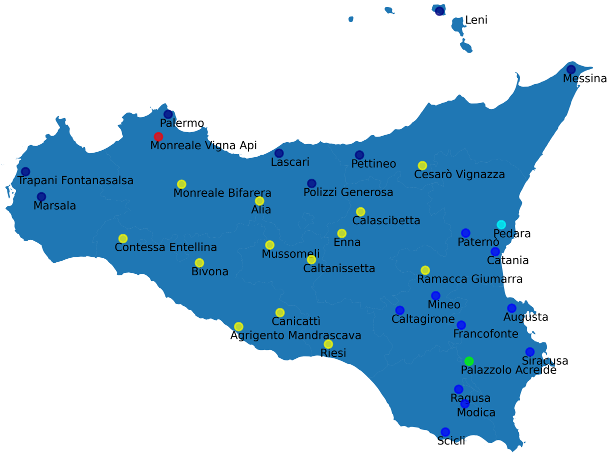

(c) Euclidean metrics and C.B

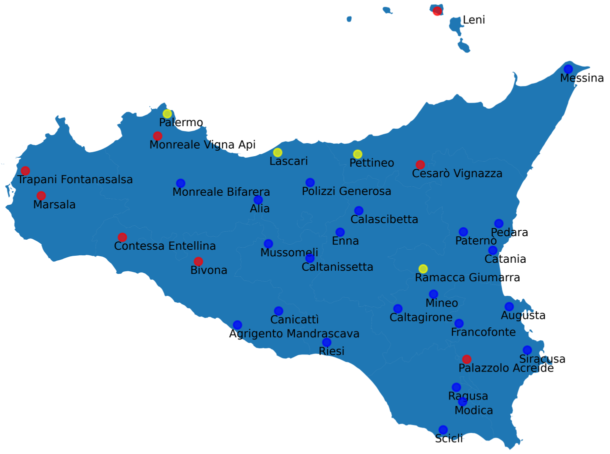

(d) Correlation metrics and C.B

Fig. S13. Annual case - 2012
Panel a: The principal cluster is reported in blue. **Panel b**: The two clusters are reported in red and blue. **Panel c**: The three principal clusters are reported in blue, yellow and dark blue. **Panel d**: The three clusters are reported in blue, red and yellow

.

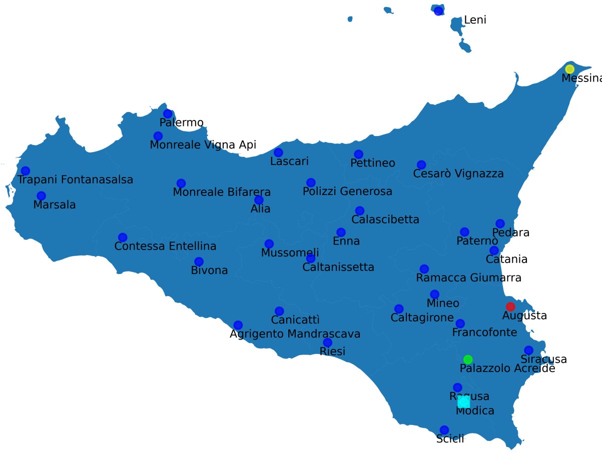

(a) Euclidean metrics and C.A

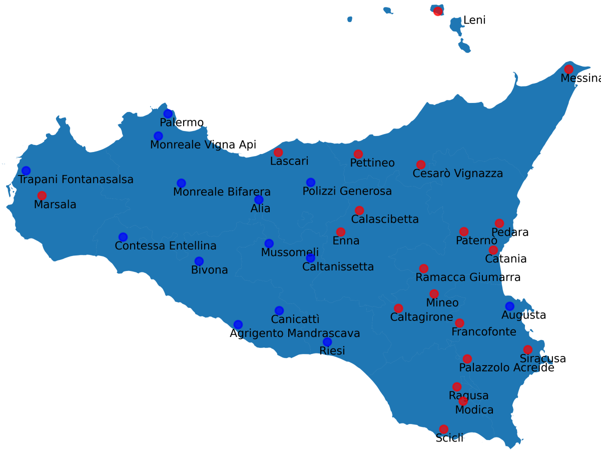

(b) Correlation metrics and C.A

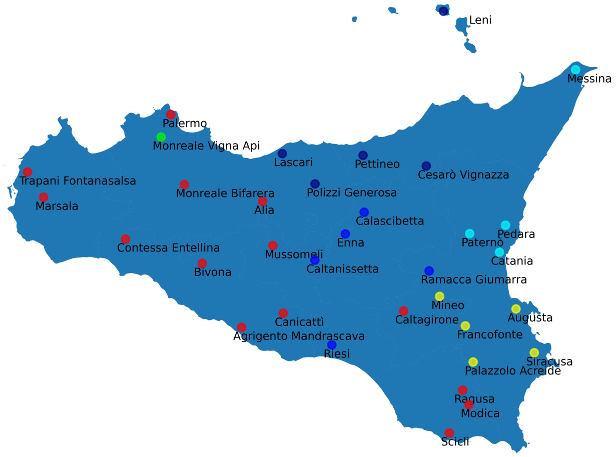

(c) Euclidean metrics and C.B

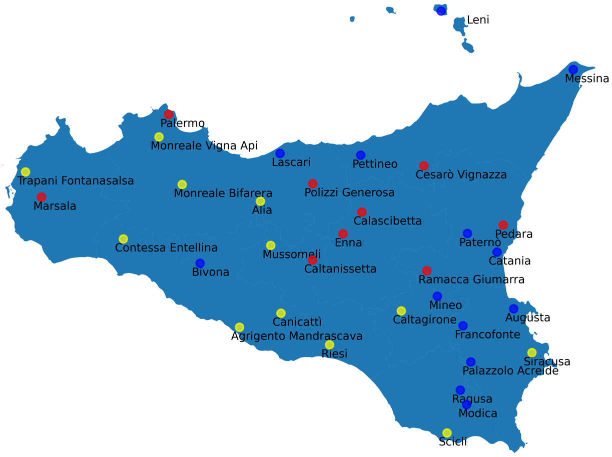

(d) Correlation metrics and C.B

Fig. S14. Annual case - 2013
Panel a: The principal cluster is reported in blue. **Panel b**: The two clusters are reported in red and blue**Panel c**: The five principal clusters are reported in red, blue, dark blue, light blue and yellow.**Panel d**: The three clusters are reported in red, blue and yellow.

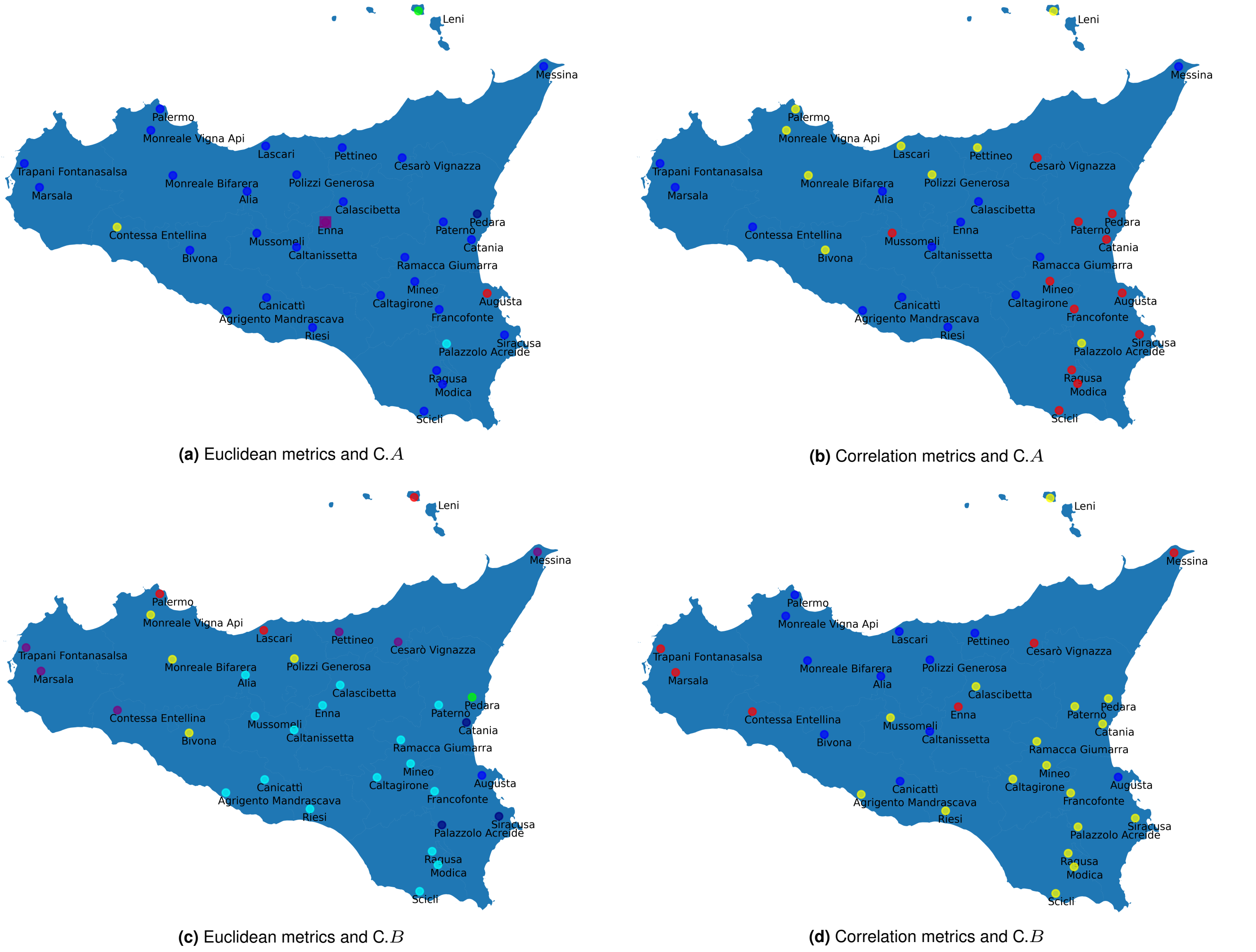

(a) Euclidean metrics and C.A

(b) Correlation metrics and C.A

(c) Euclidean metrics and C.B

(d) Correlation metrics and C.B

Fig. S15. Annual case - 2014

Panel a: The principal cluster is reported in blue. **Panel b**: The three clusters are reported in red, blue and yellow. **Panel c**: The five principal clusters are reported in light blue, yellow, purple, blue and red. **Panel d**: The three clusters are reported in red, blue and yellow.

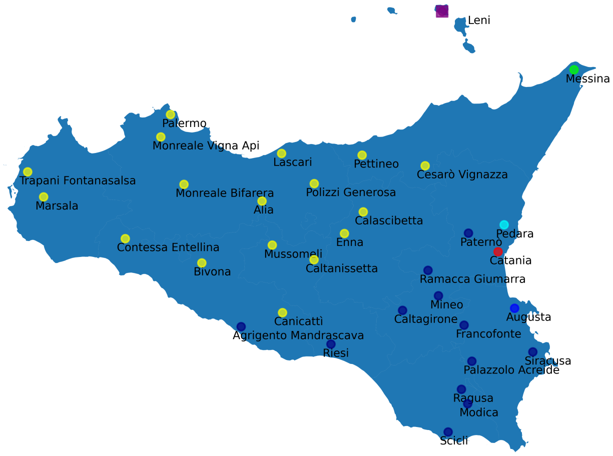

(a) Euclidean metrics and C.A

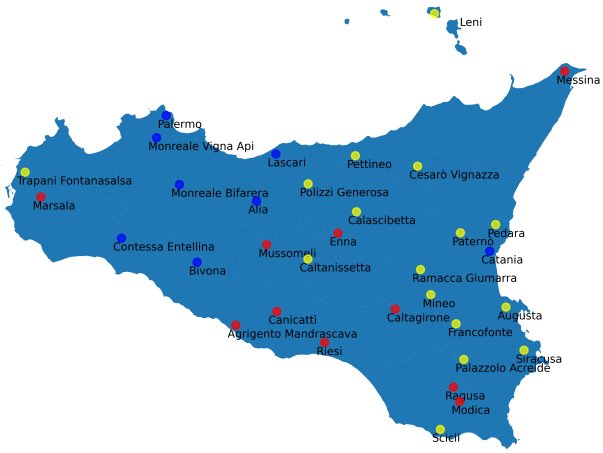

(b) Correlation metrics and C.A

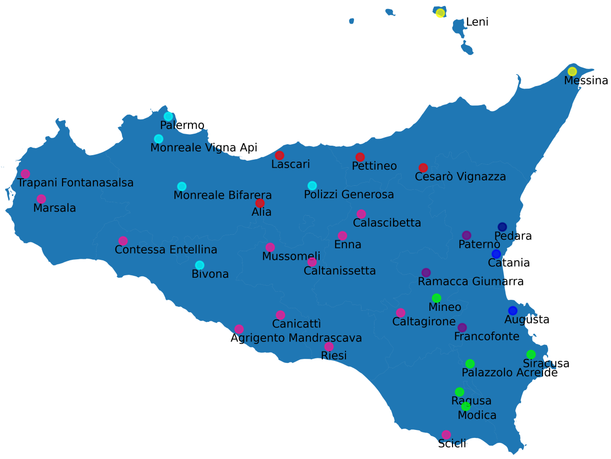

(c) Euclidean metrics and C.B

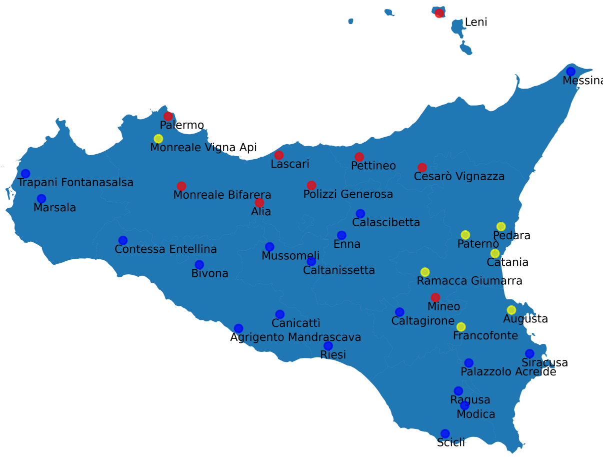

(d) Correlation metrics and C.B

Fig. S16. Annual case - 2015

Panel a: The two main clusters are reported in yellow and dark blue. **Panel b**: The three clusters are reported in red, blue and yellow. **Panel c**: The seven principal clusters are reported in pink, purple, green, blue, light blue, red and yellow **Panel d**: The three clusters are reported in red, blue and yellow.

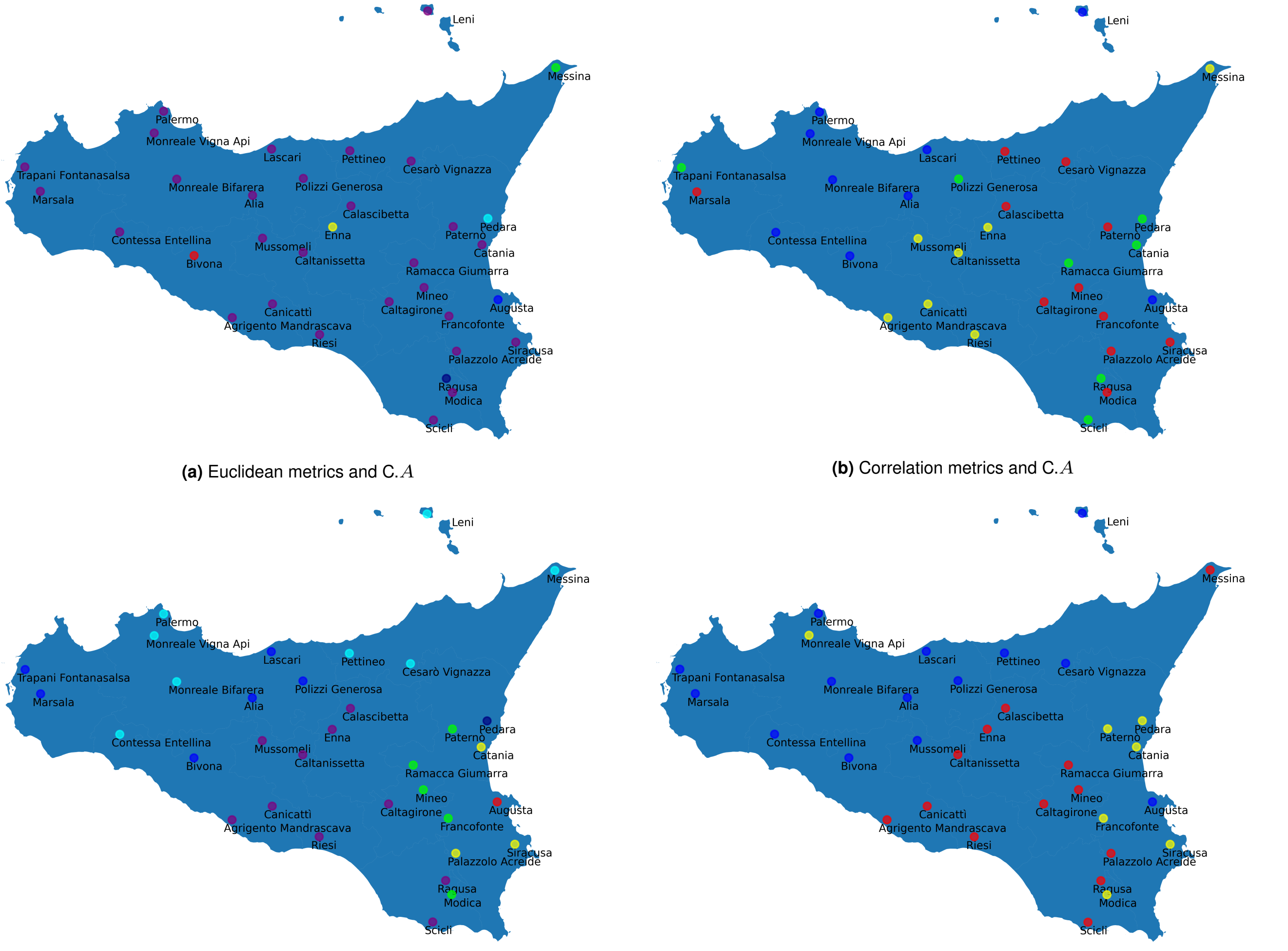

(a) Euclidean metrics and C.A

(b) Correlation metrics and C.A

(c) Euclidean metrics and C.B

(d) Correlation metrics and C.B

Fig. S17. Annual case - 2016

Panel a: The principal cluster is reported in purple. **Panel b**: The four clusters are reported in blue, red, yellow and green. **Panel c**: The five principal clusters are reported in purple, blue, light blue, green and yellow. **Panel d**: The three clusters are reported in blue, red and yellow.

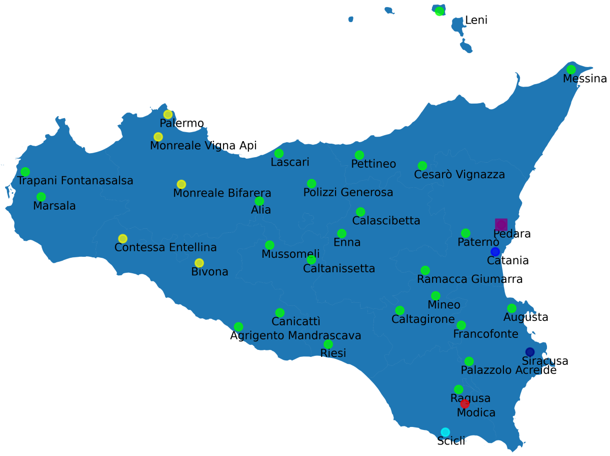

(a) Euclidean metrics and C.A

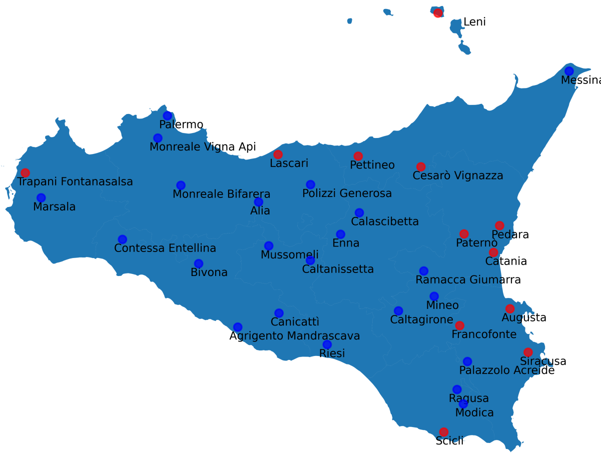

(b) Correlation metrics and C.A

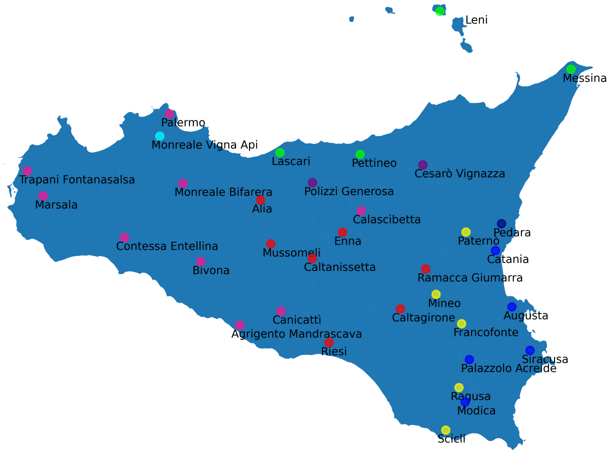

(c) Euclidean metrics and C.B

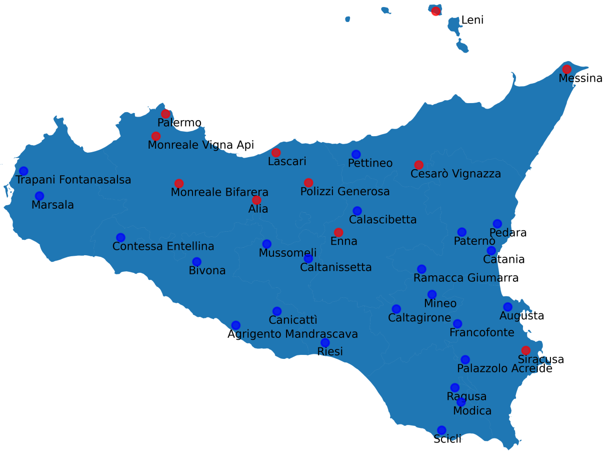

(d) Correlation metrics and C.B

Fig. S18. Annual case - 2017

Panel a: The two main clusters are reported in green and yellow. **Panel b**: The two clusters are reported in red and blue. **Panel c**: The six main clusters are reported in pink, red, yellow, blue, purple and green. **Panel d**: The two clusters are reported in red and blue.

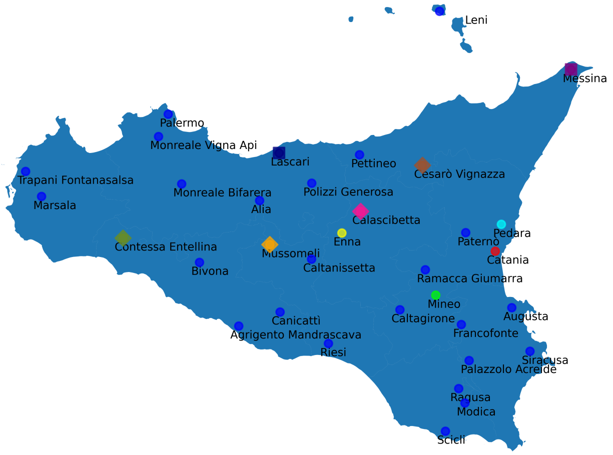

(a) Euclidean metrics and C.A

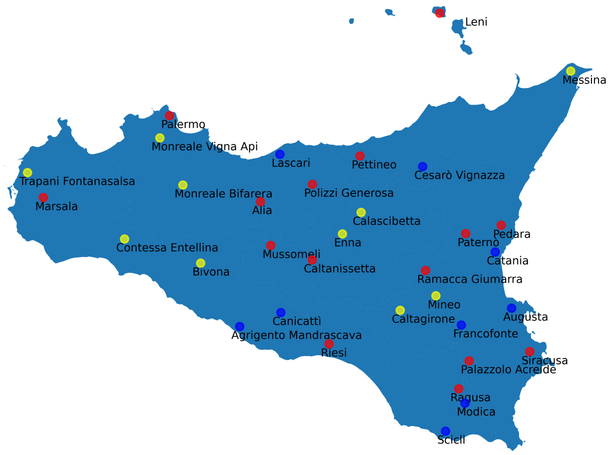

(b) Correlation metrics and C.A

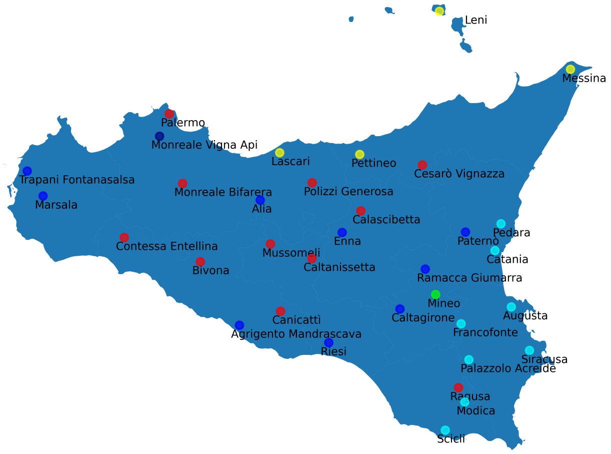

(c) Euclidean metrics and C.B

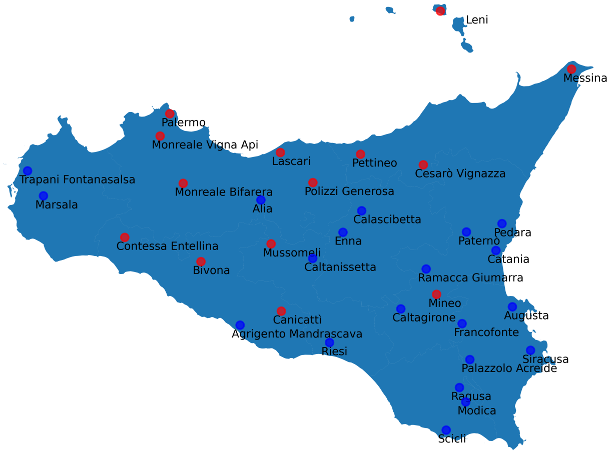

(d) Correlation metrics and C.B

Fig. S19. Annual case - 2018
Panel a: The principal cluster is reported in blue. **Panel b**: The three clusters are reported in red, blue and yellow. **Panel c**: The four main clusters are reported in red, blue, light blue and yellow.**Panel d**: The two clusters are reported in red and blue.

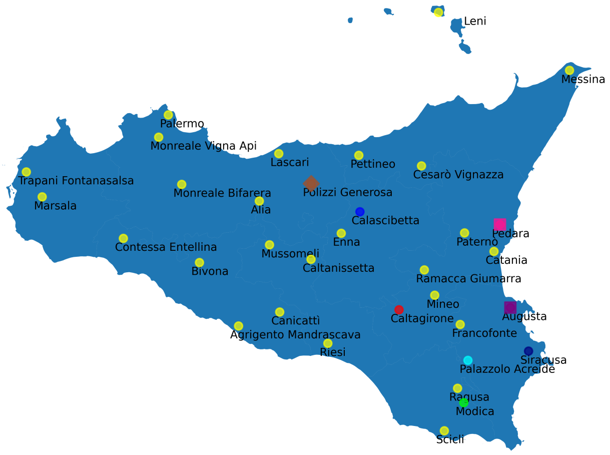

(a) Euclidean metrics and C.A

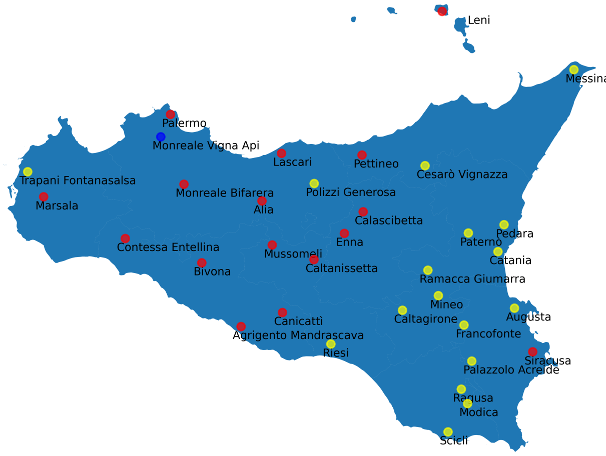

(b) Correlation metrics and C.A

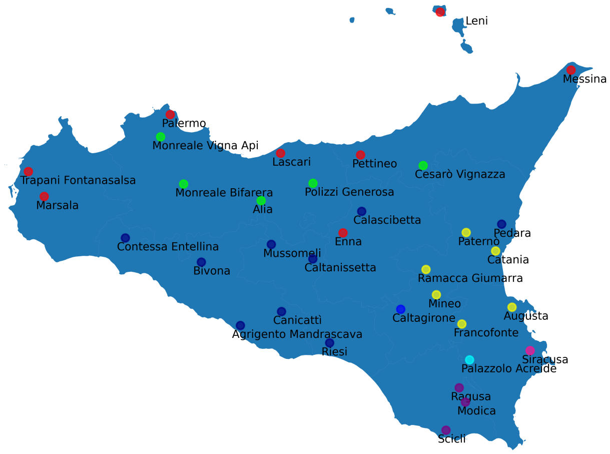

(c) Euclidean metrics and C.B

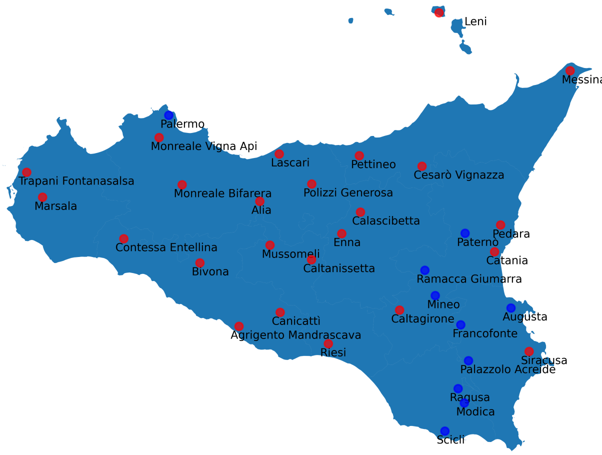

(d) Correlation metrics and C.B

Fig. S20. Annual case - 2019

Panel a: The principal cluster is reported in yellow. **Panel b**: The two main clusters are reported in red and yellow. **Panel c**: The five main clusters are reported in green, yellow, dark blue, purple and red. **Panel d**: The two clusters are reported in red and blue.

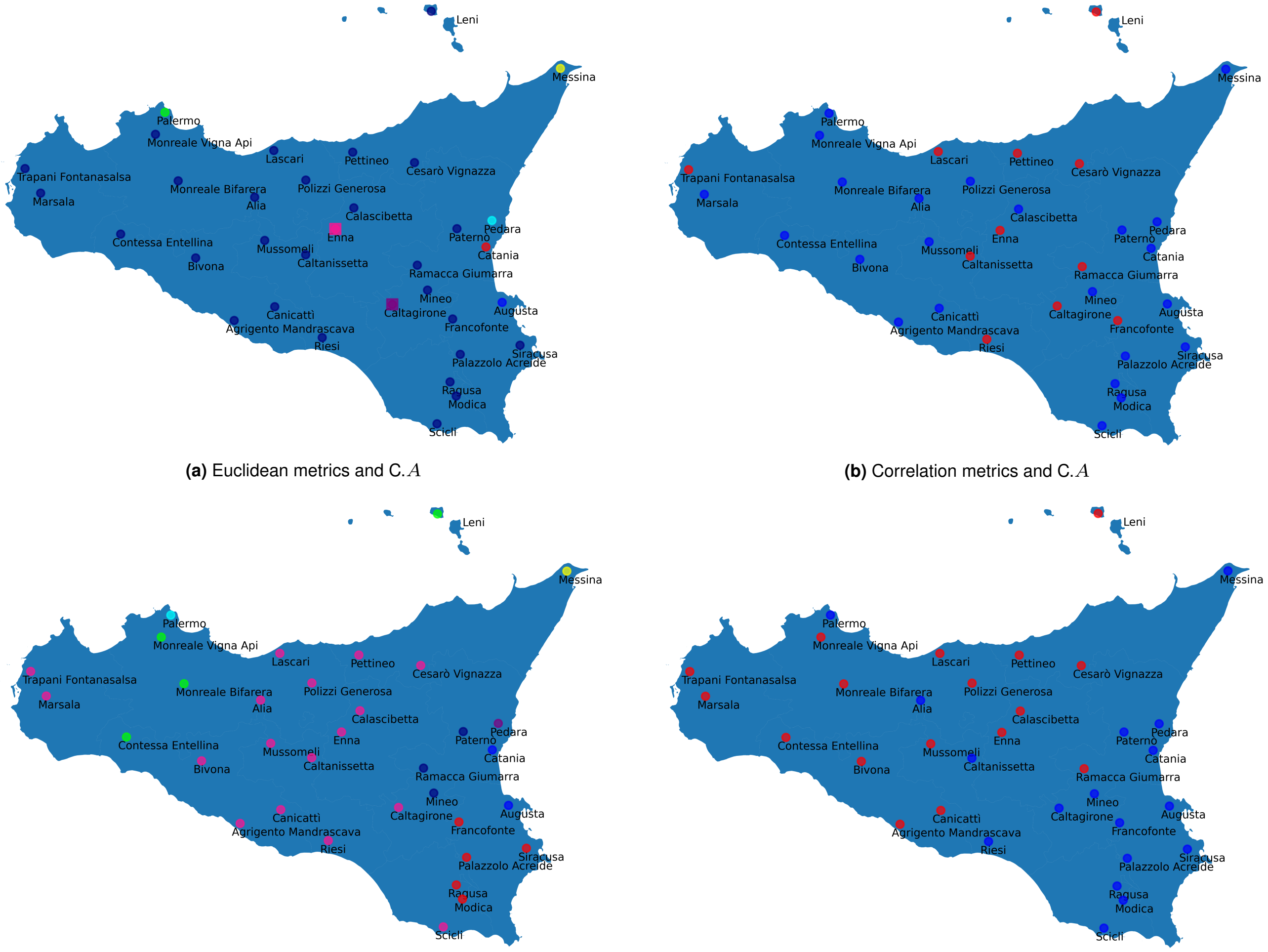

(a) Euclidean metrics and C.A

(b) Correlation metrics and C.A

(c) Euclidean metrics and C.B

(d) Correlation metrics and C.B

Fig. S21. Annual case - 2020

Panel a: The main cluster is reported in dark blue. **Panel b**: The two clusters are reported in red and blue. **Panel c**: The five main clusters are reported in green, pink, red, blue and dark blue. **Panel d**: The two clusters are reported in red and blue.

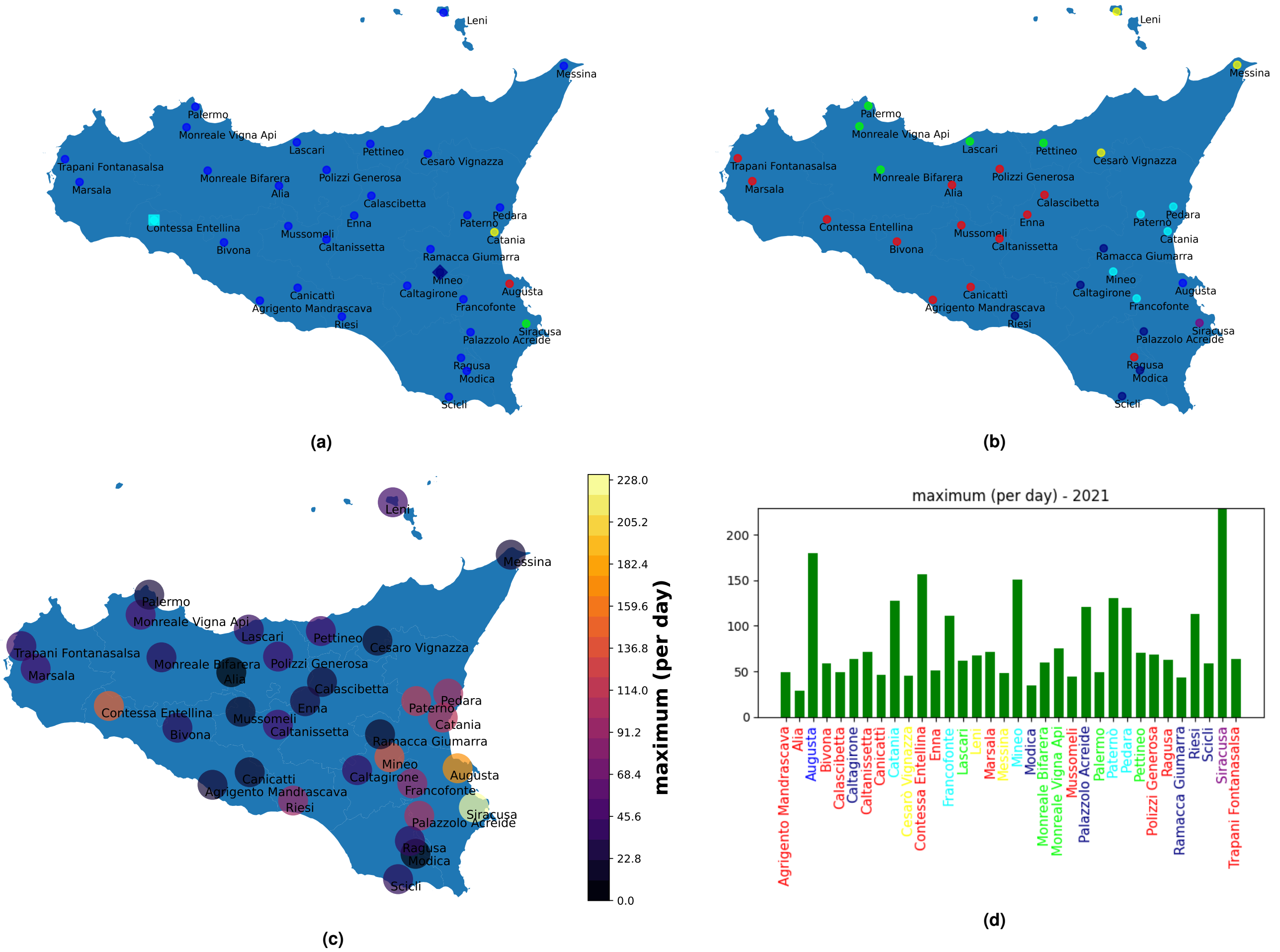

Fig. S22. Annual case for 2021 - Euclidean metrics. Different colors represent different clusters, both in the maps and in the histogram. **(a)** C.A. The principal cluster is coloured blue. Square and diamond points indicate clusters obtained by the second and the third iteration of the algorithm, respectively. **(b)** C.B. The five principal clusters are coloured red, green, blue, light blue and yellow. **(c)** Heat-map of the *md* indicator for both C.A and C.B. **(d)** Histogram of the *md* indicator with labels coloured as the C.B clustering.

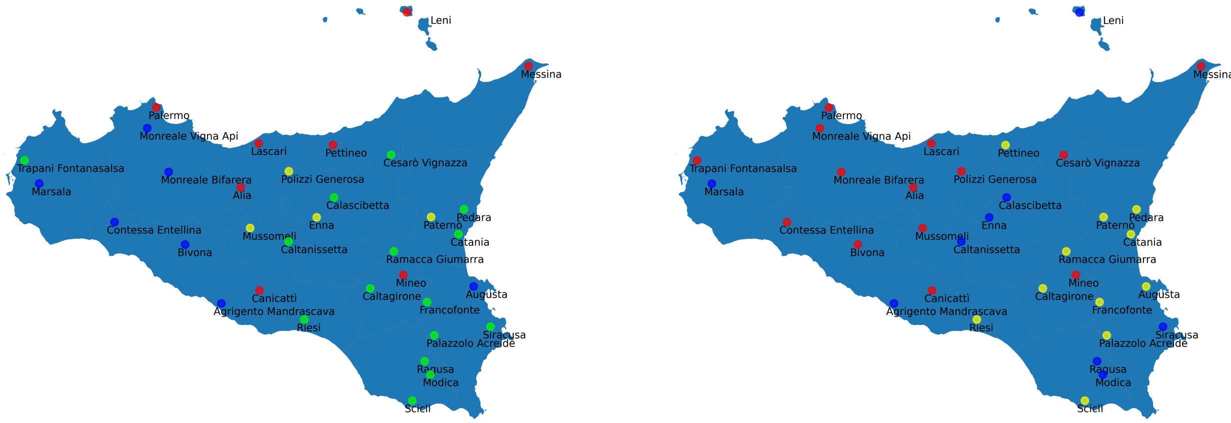

(a) **(b)**

Fig. S23. Annual case - 2021 - Correlation metrics. **(a)** C.A. The colours of the four clusters are blue, red, yellow and green. **(b)** C.B. The three clusters are coloured red, blue and yellow.
